# The reaction mechanism for glycolysis side product degradation by Parkinson’s disease–linked DJ-1

**DOI:** 10.1083/jcb.202411078

**Published:** 2025-06-04

**Authors:** Aiko Watanabe, Shizuka Ogiwara, Mirei Saito, Masaki Mishima, Masahiro Yamashina, Ryuichiro Ishitani, Yutaka Ito, Keiji Tanaka, Fumika Koyano, Koji Yamano, Hidetaka Kosako, Yoshitaka Moriwaki, Noriyuki Matsuda

**Affiliations:** 1 Department of Biomolecular Pathogenesis, Division of Advanced Pathophysiological Science, Medical Research Laboratory, Institute of Integrated Research, Institute of Science Tokyo, Tokyo, Japan; 2 Department of Molecular Biophysics, School of Pharmacy, Tokyo University of Pharmacy and Life Sciences, Tokyo, Japan; 3 Department of Chemistry, School of Science, Institute of Science Tokyo, Tokyo, Japan; 4 Department of Computational Drug Discovery and Design, Division of Biological Data Science, Medical Research Laboratory, Institute of Integrated Research, Institute of Science Tokyo, Tokyo, Japan; 5 Department of Chemistry, Tokyo Metropolitan University, Tokyo, Japan; 6 Laboratory of Protein Metabolism, Tokyo Metropolitan Institute of Medical Science, Tokyo, Japan; 7 Division of Cell Signaling, Fujii Memorial Institute of Medical Sciences, Institute of Advanced Medical Sciences, Tokushima University, Tokushima, Japan; 8 Intracellular Quality Control Project, Tokyo Metropolitan Institute of Medical Science, Tokyo, Japan

## Abstract

*DJ-1/PARK7* is the causative gene for hereditary recessive Parkinson’s disease. Recent studies have reported that DJ-1 hydrolyzes cyclic 3-phosphoglyceric anhydride (cPGA), a highly reactive metabolite. However, the molecular mechanisms underlying cPGA hydrolase activity have yet to be fully elucidated. To gain a more comprehensive understanding of this activity in DJ-1, we performed molecular simulations that predicted how DJ-1 recognizes and hydrolyzes cPGA. The accuracy of these structural predictions was validated through systematic mutational analyses exemplified by loss of activity with the A107P mutation. Although DJ-1 possesses both cPGA hydrolase and α-oxoaldehyde hydratase activities in vitro, we confirmed that DJ-1 dysfunction caused an increase in cPGA-derived modifications but had no effect on α-oxoaldehyde–derived modifications in cells. Importantly, A107 and P158, pathogenic missense mutation sites found in Parkinson’s disease patients, are critical for cPGA hydrolysis both in vitro and in cells. The evidence-based catalytic mechanism for DJ-1 hydrolysis of cPGA that we propose here explains their pathophysiological significance.

## Teaser

Although DJ-1/PARK7 has been reported to function as a cPGA hydrolase, the molecular mechanisms have yet to be fully elucidated. In this study, the authors combine in silico molecular modeling with cell-free experimental analyses of DJ-1 mutants and present an evidence-based mechanism for DJ-1–mediated cPGA hydrolysis and its pathophysiological significance.

## Introduction

*DJ-1/PARK7* is a causative gene for recessive familial Parkinson disease (hereditary Parkinsonism) (Bonifati et al., 2003). DJ-1 is thought to function as an anti-oxidative stress factor that plays an important role in protecting cells from reactive oxygen species and mitochondrial damage (Kahle et al., 2009; Taira et al., 2004; Wilson, 2011). However, despite many reports, the biochemical function of DJ-1 has yet to be fully determined. Indeed, DJ-1 has been variously reported as a redox-regulated molecular chaperone, a peroxiredoxin-like peroxidase, a transcriptional regulator, an RNA-binding protein, a deglycase, a glyoxalase, an esterase, a cysteine protease, and a cyclic 3-phosphoglyceric anhydride (cPGA) hydrolase. It has also been proposed to be a binding partner of Daxx, apoptosis signal–regulating kinase 1, and p53. Because of this complexity, the biochemical function of DJ-1 remains to be fully elucidated ([Sun and Zheng, 2023; Wilson, 2011] and references therein).

Despite the ambiguity underlying the molecular function of DJ-1, two factors informed our decision to focus our study on the cPGA hydrolase activity. First, the enzymatic activity aligns with the DJ-1 molecular structure. DJ-1/PfpI/Hsp31 family proteins are characterized by a well-conserved nucleophilic cysteine (Cys106 in human DJ-1) that is equivalent to the catalytic cysteine in cysteine proteases and the catalytic serine in α/β hydrolases. Although the typical catalytic triad structure [Cys/Ser-His(base)-Asp(acid)] is not conserved in DJ-1 itself, it exists in DJ-1 relatives such as HchA. Because α/β hydrolase fold enzymes typically hydrolyze C-O or C-N bonds (Dimitriou et al., 2019; Nardini and Dijkstra, 1999; Rauwerdink and Kazlauskas, 2015) and cysteine proteases catalyze peptide bond hydrolysis, we speculated that the DJ-1 nucleophilic cysteine (C106) plays a similar hydrolytic role. In fact, all the enzymatic reactions described above are initiated by nucleophilic attack on the carbonyl groups of the respective substrates. The cPGA hydrolytic reaction also fits these criteria, which supports a catalytic role for DJ-1 in cPGA hydrolysis. Second, the esterase activity examined in vitro might reflect the physiological cPGA hydrolase activity of DJ-1. We previously analyzed DJ-1 esterase activity in detail (Watanabe et al., 2024) and found that in certain aspects its activity as an esterase was more evident than as a methylglyoxalase (e.g., its *k*_cat_ as an esterase was 50 times higher than as a glyoxalase). However, that study raised important questions about the physiological relevance of the DJ-1 substrates assayed. While glyoxal and methylglyoxal (MGO) may be genuine physiological substrates because both are present in cells, 4-nitrophenyl acetate is an artificial esterase substrate that does not occur in cells. Consequently, it is unclear how the in vitro esterase activity of DJ-1 manifests in vivo. Given that cPGA is a highly reactive cyclic ester, it is interesting if the in vitro esterase activity might reflect physiological cPGA hydrolysis activity. Indeed, even though there is no precedent report of cPGA hydrolase activity in α/β hydrolase fold proteins, cyclic-ester hydrolase activity (i.e., lactonase activities), which is reminiscent of cPGA hydrolase activity, has been reported in several α/β hydrolase fold proteins (Rauwerdink and Kazlauskas, 2015).

Despite these lines of evidence, comprehensive analysis of the molecular mechanisms underlying cPGA hydrolysis activity has lagged behind due to technical difficulties associated with the instability of cPGA as a substrate. Indeed, mutational analyses to identify amino acid residues critical for cPGA hydrolase activity have been limited to C106, and no structural models of either the cPGA–DJ-1 complex or the hydrolytic mechanism have been proposed. In this paper, we combined molecular simulations with biochemical analyses to gain mechanistic insights into DJ-1 hydrolysis of cPGA. Moreover, cell-free assays that reconstituted endogenous DJ-1 function and mass spectrometry analyses of cultured cells suggest that cPGA hydrolytic activity rather than glyoxalase activity is the genuine in vivo function of DJ-1. These results provide an important clue for understanding the molecular role that DJ-1 has in the suppression of early onset familial Parkinson’s disease.

## Results

### cPGA hydrolase activity of prokaryotic DJ-1 homologs

**Figure 1.**
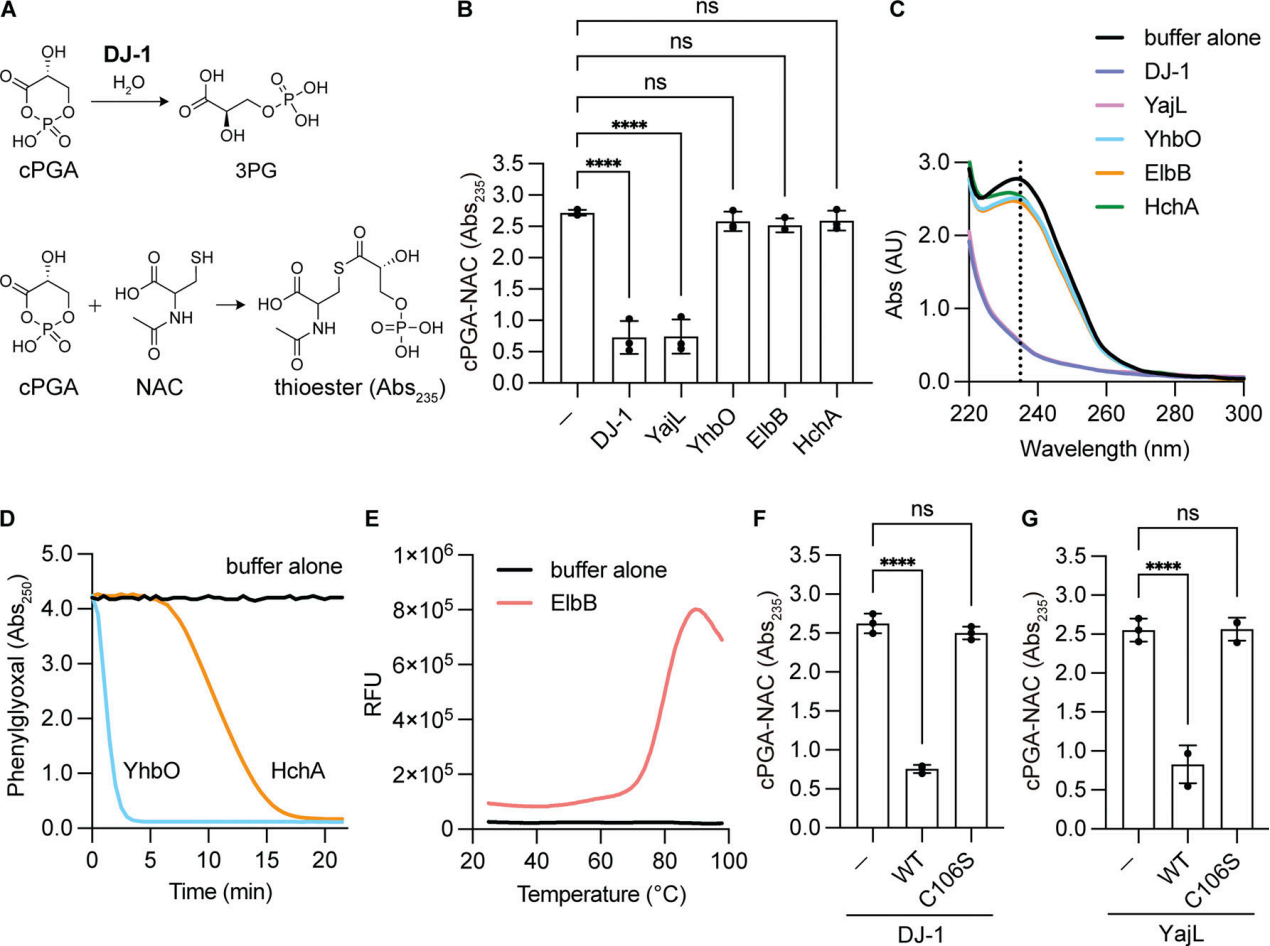
**cPGA hydrolase activity is present in YajL but not the other prokaryotic DJ-1 homologs. (A)** Reaction mechanism for DJ-1 hydrolase conversion of cPGA to 3PG. Since Abs_235_ indicates thioester formation following cPGA reaction with N-acetyl-L-cysteine (NAC), cPGA consumption by DJ-1 can be monitored by a reduction in Abs_235_. **(B)** cPGA hydrolase activity was not observed in YhbO, ElbB, or HchA but was present in YajL. cPGA was incubated with DJ-1, YajL, YhbO, ElbB, or HchA for 3 min, followed by reaction with NAC. A reduction in Abs_235_ is indicative of cPGA hydrolase activity (*n* = 3). **(C)** Absorbance spectra of reaction mixtures containing cPGA and WT DJ-1 (blue), YajL (pink), YhbO (light blue), ElbB (orange), or HchA (green) followed by incubation with NAC. The black line corresponds to buffer alone. **(D)** Phenylglyoxalase activities of YhbO (light blue) and HchA (orange). The PGO-derived Abs_250_ decreased following incubation with YhbO or HchA. The black line corresponds to buffer alone. **(E)** The ElbB thermal shift melt curve confirmed that the enzyme was correctly folded. The black line corresponds to buffer alone. **(F and G)** A decrease in Abs_235_ is indicative of cPGA consumption following incubation for 3 min with WT or the C106S mutant of DJ-1 (F) or YajL (G) (all *n* = 3). Data shown in C–E are representative of three individual experiments. Data in B, F, and G are the mean ± SD of three experiments. *P < 0.05 using one-way ANOVA with Dunnett’s multiple comparisons test (B, F, and G). RFU, relative fluorescence unit.

cPGA is a reactive cyclic ester that causes nonspecific acylation of intracellular proteins and metabolites (Moellering and Cravatt, 2013). This reactive carbonyl compound has attracted attention recently because the Parkinson disease causative factor DJ-1 has been reported to protect proteins/metabolites from cPGA-mediated modification by catalyzing the conversion of cPGA to 3-phosphoglycerate (3PG) (Akhmadi et al., 2024; Heremans et al., 2022) (Fig. 1 A, top panel). DJ-1 is a highly conserved protein with homologs present in bacterial genomes. The *Escherichia coli* genome encodes four DJ-1 homologs, YajL, HchA, YhbO, and ElbB (Abdallah et al., 2016; Lee et al., 2016; Subedi et al., 2011). If cPGA hydrolase activity is a genuine function of DJ-1, it is reasonable to hypothesize that the enzymatic activity is also evolutionarily conserved. While YajL possesses cPGA hydrolase activity (Akhmadi et al., 2024), it is unknown if other prokaryotic DJ-1 homologs also possess this enzymatic activity. We thus first investigated evolutionary conservation of the hydrolytic activity among prokaryotic DJ-1 homologs.

We expressed and purified four *E. coli* DJ-1 homologs (YajL, HchA, YhbO, and ElbB) as reported previously (Watanabe et al., 2024) and assessed their cPGA hydrolase activity. Each purified protein was reacted with cPGA followed by incubation with N-acetylcysteine. Reacting N-acetylcysteine with cPGA yields a characteristic Abs235 peak (Fig. 1 A, bottom panel). The Abs235, however, will disappear if cPGA is converted to 3PG. A change in Abs235 thus can be used as an indicator of cPGA hydrolase activity (Akhmadi et al., 2024). Among the four DJ-1 homologs tested, only YajL exhibited cPGA hydrolase activity comparable with that of DJ-1 (Fig. 1, B and C). In contrast, the other three homologs—HchA, YhbO, and ElbB—did not demonstrate any enzymatic activity for cPGA (Fig. 1, B and C). This finding indicates functional divergence of the four DJ-1 homologs, with only YajL retaining cPGA hydrolase activity. Previously, we performed molecular evolutionary analysis of DJ-1 family proteins and generated phylogenetic profiles of DJ-1 and its bacterial homologs (Watanabe et al., 2024). Interestingly, the enzymatic activities are consistent with the phylogenetic profiles. While YajL is the *E. coli* counterpart of human DJ-1 and ElbB is the counterpart of human GATD3 (and to a lesser extent DJ-1), there is no corresponding gene for either YhbO or HchA in the human genome.

To rule out the possibility that the non-active DJ-1 homologs were inactive because of protein misfolding, we assessed the α-oxoaldehyde hydratase activity (glyoxalase III activity) of HchA and YhbO (Abdallah et al., 2016; Lee et al., 2016; Subedi et al., 2011). Glyoxalase III activity was clearly observed for both (Fig. 1 D), indicating that the absence of cPGA hydrolase activity reflected functional loss rather than incorrect folding. Since ElbB lacks this enzymatic activity, we monitored its folding state via a thermal shift assay (Lo et al., 2004; Queliconi et al., 2021; Semisotnov et al., 1991), in which the protein of interest is incubated with a specialized dye, and its fluorescence is measured in relation to increasing temperature. When the protein unfolds, the exposed hydrophobic surface binds the dye, resulting in increased fluorescence. Unfolding temperatures, which are analogous to melting temperatures (referred to as Tm), indicate the maximum value of the first derivative of the relative fluorescence unit as a function of temperature (dRFU/dT). Evidence for ElbB unfolding was observed around 70°C (Tm = 79.9°C) (Fig. 1 E), suggesting that it was initially folded and that the lack of cPGA hydrolase activity was due to a loss of function. Thus, of the four bacterial homologs assayed, the DJ-1 hydrolytic activity was only conserved in YajL. Moreover, this activity was eliminated following mutation of the catalytic cysteine (Cys106) in both DJ-1 and YajL (Fig. 1, F and G), confirming that the activity observed was derived from the recombinant proteins. Taken together, these results highlight the unique cPGA hydrolase activity of YajL among the four homologs.

### Molecular simulation of DJ-1 and YajL

**Figure 2.**
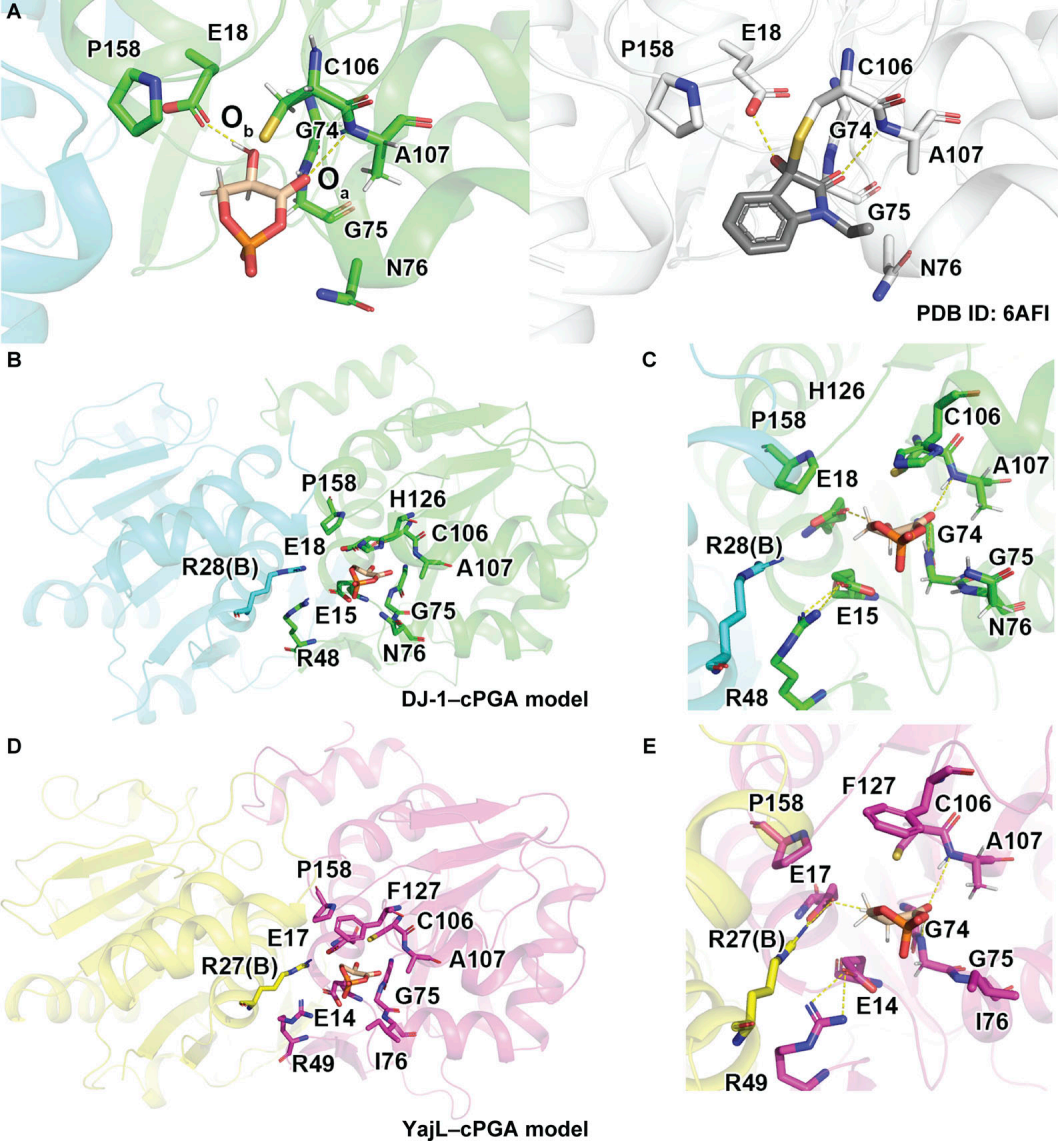
**Molecular simulation of DJ-1 and YajL complexed with cPGA. (A)** Molecular model of how cPGA (orange) is recognized by DJ-1. The DJ-1–cPGA complex (left) and the initial structural template of DJ-1 (right) conjugated with the covalent inhibitor 1-ethylindole-2,3-dione (gray) are shown in parallel. **(B)** DJ-1–cPGA complex. Amino acids in DJ-1 (E15, E18, G74, G75, N76, C106, A107, H126, and P158) that contact cPGA (orange) are highlighted as is R48, which forms a salt bridge with E15, and R28 from another protomer in the DJ-1 dimer forms a salt bridge with E18. **(C)** Magnified view of the DJ-1 and cPGA-binding site. **(D)** Molecular model of YajL complexed with cPGA. Amino acids (E14, E17, G74, G75, I76, C106, A107, F127, and P158) that form the cPGA-binding pocket are highlighted as is R27, which forms a salt bridge with E17. R27 is derived from another protomer. **(E)** Magnified view of the YajL and cPGA-binding site.

To fully elucidate the molecular mechanisms underlying cPGA hydrolase activity, we next evaluated the DJ-1 and YajL structures complexed with cPGA. Crystal structures of DJ-1 and YajL have already been determined (Honbou et al., 2003; Huai et al., 2003; Lee et al., 2003; Tao and Tong, 2003; Wilson et al., 2003, 2005) as have structures of DJ-1 with different covalent inhibitors that mimic substrate–enzyme interactions (Choi et al., 2014; Tashiro et al., 2018), such as a crystal structure of DJ-1 complexed with 1-ethylindole-2,3-dione (PDB ID: 6AFI). We previously showed that nucleophilic C106 attacked the carbon of carbonyl group of 1-ethylindole-2,3-dione when the inhibitor forms covalent conjugation with DJ-1 (Tashiro et al., 2018). This mechanism seemed similar to that proposed for DJ-1 hydrolysis of cPGA (see Discussion). We thus used this crystal structure (DJ-1 conjugated with 1-ethylindole-2,3-dione) as a template to model the DJ-1–cPGA complex (Fig. 2 A) and conducted molecular dynamics (MD) simulations to evaluate its stability. In this model, we assumed that the cPGA-binding site coincides with that of other substrates. In particular, the oxygen atoms of the keto and hydroxy groups in cPGA overlap with those of known substrates with similar chemical structures. Our subsequent MD simulation revealed that the position of cPGA in the putative binding pocket was stable throughout the 50-ns simulation period and that the pocket is formed by E15, E18, G74, G75, N76, C106, A107, H126, P158, and R28 from another protomer in the DJ-1 dimer (Fig. 2, B and C; and Video 1). We also constructed a cPGA-bound model with the YajL structure, the backbone of which is almost identical to DJ-1 with an Cα RMSD value of 2.01 Å (Matsuda et al., 2017; Wilson et al., 2005). The corresponding binding pocket residues in YajL consisted of E14, E17, G74, G75, I76, C106, A107, F127, P158, and R27 from another protomer in the YajL dimer (Fig. 2, D and E).

**Video 1.** **This movie shows a 50-ns MD simulation of DJ-1 in complex with cPGA.** Orange and green represent cPGA and the cPGA-interacting protomer of the DJ-1 dimer, respectively. Cyan indicates the other protomer of the DJ-1 dimer, which provides residue R28 to form the cPGA-binding pocket. The 50-ns simulation is played over 8 s. Fig. 2, B and C; and Fig. 4 A contain related video stills or images.

**Figure 3.**
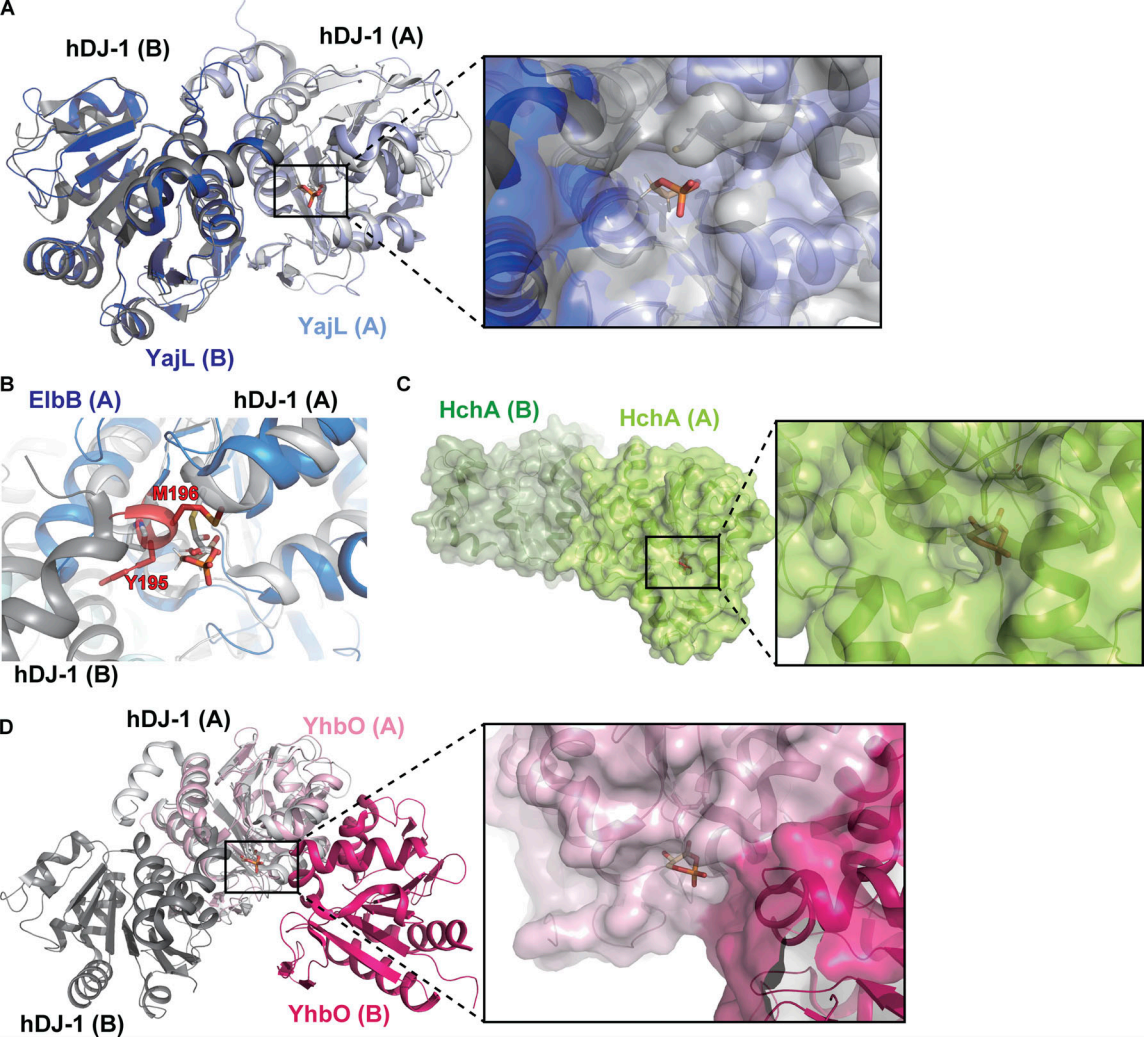
**Dimeric structures of ElbB, HchA, and YhbO weaken cPGA binding. (A)** Overlaid image of the DJ-1 or YajL molecular model complexed with cPGA. Magnified view shows the cPGA-binding site (orange) with the surface structure of DJ-1 (gray) and YajL (blue). **(B)** Molecular model of the cPGA-binding site in ElbB (blue). The helix-forming residues of ElbB that interfere with cPGA binding are highlighted in red. **(C)** Molecular model of the HchA–cPGA complex with a magnified view. **(D)** Overlaid image of DJ-1 or YhbO complexed with cPGA. Magnified view shows the surface structure of YhbO in proximity to the cPGA-binding site.

Our proposed model for cPGA recognition by DJ-1 and YajL (Fig. 2) can explain why ElbB, YhbO, and HchA failed to catalyze cPGA hydrolysis (Fig. 1 B). The dimeric complex of YajL (PDB ID: 2AB0) closely resembles that of DJ-1, with an almost identical arrangement of residues around the binding pocket (Fig. 3 A). In contrast, the structures of ElbB (PDB ID: 1VHQ) and HchA (PDB ID: 1IZY) differ from DJ-1/YajL. For instance, a helical segment unique to the ElbB structure that comprises residues A194 to L197 interferes with the cPGA-binding pocket (Fig. 3 B), and HchA has an additional tertiary structure that covers the pocket and prevents substrate entry (Fig. 3 C). Although the amino acid composition of YhbO (PDB ID: 1OI4) is similar to DJ-1/YajL, its dimer interface differs markedly. This difference makes the binding pocket larger than the substrate, weakening its ability to effectively bind cPGA (Fig. 3 D). Thus, structural features of the DJ-1 homologs can account for the differences in their cPGA hydrolytic activity.

### R28–E18 interaction assists in cPGA recognition by DJ-1

**Figure 4.**
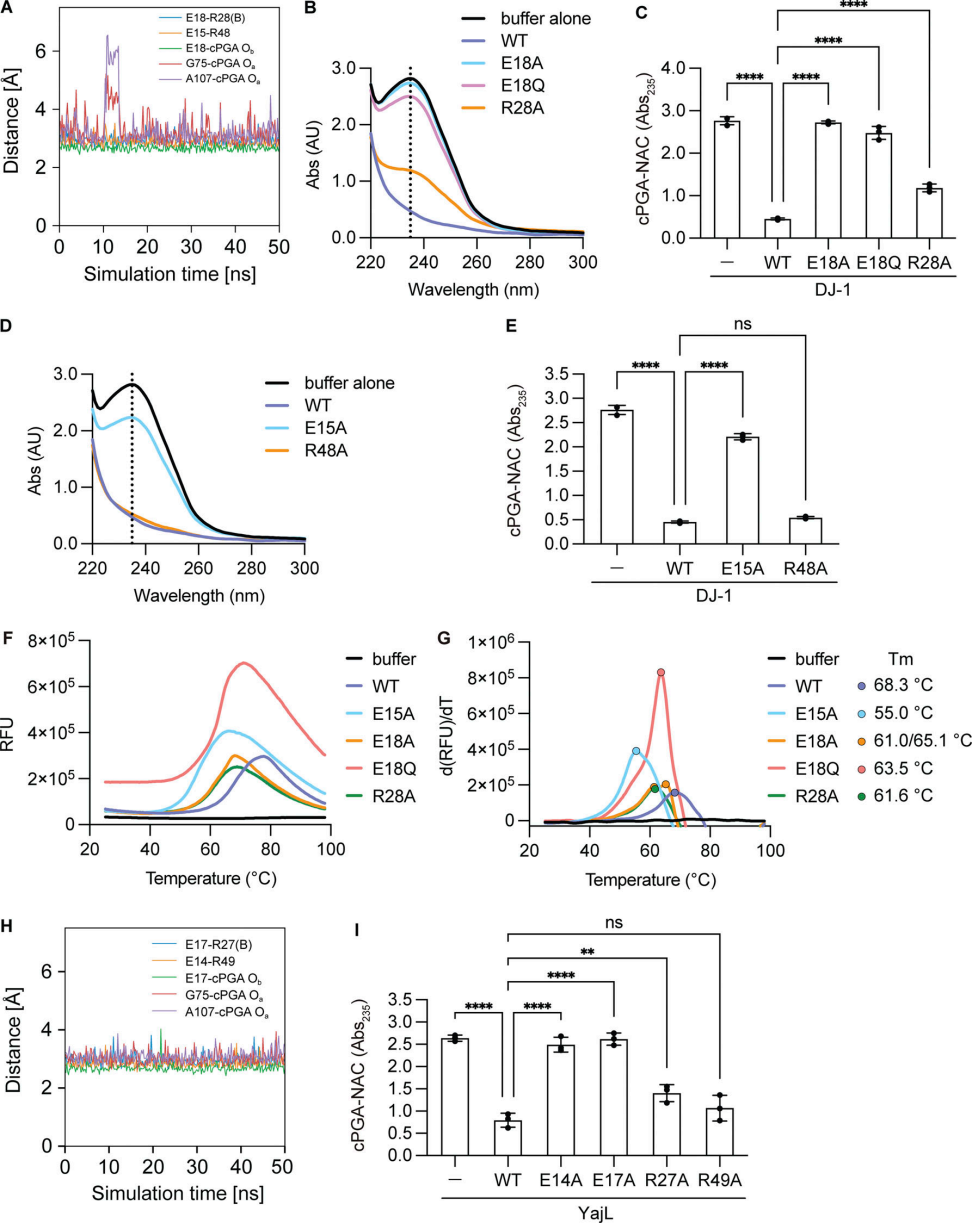
**The catalytically critical E18 is stringently regulated by R28. (A)** Distance plot of residues that form the cPGA-binding site in DJ-1 based on the unstrained 50-ns MD simulation. **(B)** Absorbance spectra of reaction mixtures containing cPGA and WT (blue), E18A (light blue), E18Q (pink), or R28A (orange) DJ-1. Initial reactions were incubated for 3 min, and then N-acetyl-L-cysteine (NAC) was added. The thioester peak at Abs_23_ decreases if the DJ-1 mutants can hydrolyze cPGA. The black line corresponds to buffer alone. **(C)** The cPGA hydrolytic activity of WT, E18A, E18Q, or R28A DJ-1 was monitored by a decrease in Abs_235_ (all *n* = 3). **(D)** Absorbance spectra are as in B following incubation with WT (blue), E15A (light blue), or R48A (orange) DJ-1. **(E)** The cPGA hydrolytic activity of WT, E15A, or R48A DJ-1 was monitored by a decrease in Abs_235_ (all *n* = 3). **(F and G)** Thermal shift denaturation curves of WT (blue), E15A (light blue), E18A (orange), E18Q (red), or R28A (green) DJ-1. Representative relative fluorescence unit (RFU) (F) and positive derivative [d(RFU)/dT] curves with melting temperatures (Tm) (G) are shown. The black line indicates the buffer control. **(H)** Distance plot of residues that form the cPGA-binding site in YajL based on the unstrained 50-ns MD simulation. **(I)** The cPGA hydrolytic activity of WT or the indicated YajL mutants was monitored by a decrease in Abs_235_ (all *n* = 3). Data shown in B, D, F, and G are representative of three individual experiments. Data in C, E, and I are the mean ± SD of three experiments. *P < 0.05 using one-way ANOVA with Dunnett’s multiple comparisons test (C, E, and I).

To gain insights into the molecular mechanism underpinnings of cPGA hydrolysis by DJ-1 and YajL, we mutated residues comprising the binding pocket and biochemically assessed changes to hydrolytic activity. We previously reported that E18 in DJ-1 and its equivalent residue (E77) in HchA are essential for methylglyoxalase and esterase activities (Matsuda et al., 2017; Watanabe et al., 2024), suggesting the importance of this residue. Based on our model, E18 forms a hydrogen bond with the hydroxy group of cPGA (Fig. 2 A, left panel) and forms a salt bridge with R28 in the other chain thorough the dimer interface (Fig. 2 C). We confirmed that these interactions were stable throughout the 50-ns simulation period (Fig. 4 A and Video 1). Enzymatic activity was completely abolished with E18A and E18Q mutations and was reduced by the R28A mutation (Fig. 4, B and C). This is consistent with our MD simulations upon the crystal structure because cPGA is located near those residues. E15 is also in proximity to cPGA and formed a salt bridge with R48 in the MD simulations (Fig. 2 C and Fig. 4 A). The E15A mutation also completely abolished activity, whereas the R48A mutation had no effect (Fig. 4, D and E). These results suggest that although both E15 and E18 comprise the substrate-binding pocket, E18 is more directly involved than E15. To interpret the mutational data appropriately, it is necessary to demonstrate that the missense mutations did not disrupt the overall DJ-1 structure. The E18A mutation was reported to have no effect on the structure (Prahlad et al., 2014), and thermal shift data demonstrated that the E15A, E18A, E18Q, and R28A mutants were folded at room temperature but started unfolding around 50°C (Tm = 68.3°C, 55.0°C, 61.0/65.1°C, 63.5°C, and 61.6°C for WT, E15A, E18A, E18Q, and R28A, respectively), confirming that the loss of enzymatic activity following mutation of E15, E18, and R28 was not the result of complete misfolding (Fig. 4, F and G).

To examine the importance of the residues from an evolutionary perspective, similar mutations were introduced in YajL, and their enzymatic activities were analyzed. Based on our MD simulations, the structural relationship between cPGA and the corresponding residues in YajL (i.e., E14, E17, R27, and R49) overlaps with the E15, E18, R28, and R48 residues in DJ-1 (Fig. 2, C and D). We confirmed that cPGA–YajL interaction was stable as distance between E17 and the hydroxy group of cPGA or a salt bridge between E17 and R27 was unchanged throughout the 50-ns simulation period (Fig. 4 H). Similar to DJ-1, the E14A and E17A mutations in YajL completely abolished cPGA hydrolytic activity, R27A significantly reduced it, and R49A had no effect (Fig. 4 I).

### C106 and G74/75 form the catalytic center and the oxyanion hole for cPGA hydrolase activity

As described above, DJ-1/PfpI/Hsp31 family proteins and α/β hydrolase superfamily proteins share a structurally related nucleophilic Ser/Cys that functions as the catalytic center (Dimitriou et al., 2019; Nardini and Dijkstra, 1999; Rauwerdink and Kazlauskas, 2015). In the case of α/β hydrolase, the nucleophilic residue attacks the substrate and forms an acyl-enzyme intermediate during catalysis. In addition, hydrogen bonds formed between the oxyanion hole and the substrate carbonyl group play a crucial role in stabilizing the enzyme-substrate intermediate that lowers the activation barrier of the nucleophilic attack process (Dimitriou et al., 2019; Nardini and Dijkstra, 1999; Rauwerdink and Kazlauskas, 2015). Numerous studies have reported that C106 of DJ-1 is essential for enzymatic activity (Akhmadi et al., 2024; Andreeva et al., 2019; Choi et al., 2023; Gao et al., 2023; Heremans et al., 2022; Lee et al., 2012; Matsuda et al., 2017; Mazza et al., 2022; Richarme et al., 2015; Vázquez-Mayorga et al., 2016; Watanabe et al., 2024). In contrast, despite G74 and G75 being highly conserved in DJ-1 family proteins, their importance in the enzymatic activity of DJ-1 has yet to be investigated. We previously demonstrated that G153 and G154 of HchA, which correspond to G74 and G75 of DJ-1, are essential for the phenylglyoxalase and methylglyoxalase activities (Watanabe et al., 2024). To our knowledge, this is the only report highlighting the importance of a double glycine motif as the oxyanion hole.

**Figure 5.**
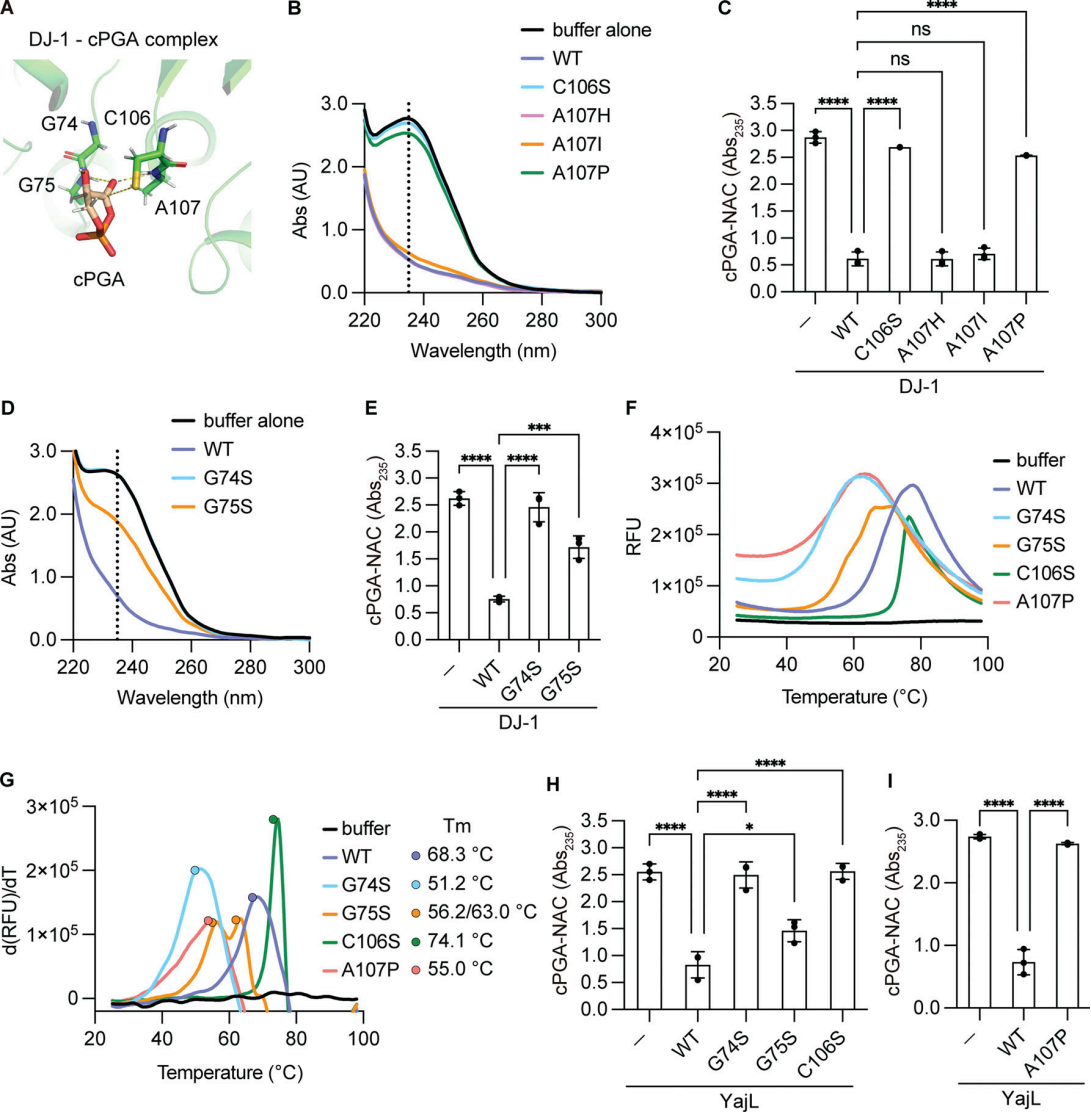
**G74–G75 comprise an oxyanion hole that supports formation of the substrate–enzyme intermediate connected via C106. (A)** Structural model depicting hydrogen bond formation of G74–G75 and A107 with the cPGA carbonyl group. **(B)** Absorbance spectra when cPGA was incubated with WT (blue), C106S (light blue), A107H (pink), A107I (orange), or A107P (green) DJ-1. Initial reactions were incubated for 3 min, and then N-acetyl-L-cysteine (NAC) was added. The black line indicates the buffer control. **(C)** The cPGA hydrolytic activity of the indicated DJ-1 mutants was monitored by a decrease in Abs_235_ (all *n* = 3). **(D)** Absorbance spectra showing consumption of cPGA by WT (blue), G74S (light blue), or G75S (orange) DJ-1 mutants. **(E)** The cPGA hydrolytic activity of the indicated DJ-1 mutants was monitored by a decrease in Abs_235_ (all *n* = 3). **(F and G)** Thermal shift denaturation curves of WT (blue), G74S (light blue), G75S (orange), C106S (green), or A107P (red) DJ-1. Representative relative fluorescence unit (RFU) (F) and positive derivative [d(RFU)/dT] curves with melting temperatures (Tm) (G) are shown. The black line indicates the buffer control. **(H and I)** cPGA consumption by WT YajL or the indicated YajL mutants was monitored as in C and E (all *n* = 3). Data shown in B, D, F, and G are representative of three individual experiments. The mean ± SD of three experiments is shown in C, E, H, and I. *P < 0.05 using one-way ANOVA with Dunnett’s multiple comparisons test (C, E, H, and I).

Our structural analysis clearly implies that the C106 thiol in DJ-1 localizes very close to the carbon atom of the cPGA carbonyl group (Fig. 5 A), suggesting that C106 attack of the carbonyl carbon to form the first tetrahedral intermediate (discussed later in Fig. 7). Consistent with previous reports (Akhmadi et al., 2024; Heremans et al., 2022), a C106S mutation completely abolished cPGA hydrolytic activity in DJ-1 (Fig. 5, B and C). More interestingly, the model predicts a hydrogen bond between the backbone nitrogen atom of A107 and the oxygen atom of the cPGA carbonyl group. We examined the significance of this bond and found that an A107P mutation eliminated activity, whereas A107H and A107I mutations had no effect (Fig. 5, B and C). Because the proline side chain specifically masks the nitrogen atom, these results emphasize the importance of this hydrogen bond. It is noteworthy that a homogenic A107P variant of DJ-1 (c.319G>C/c.319G>C as the genomic mutation) was reported as a pathogenic mutation in a 22 -year-old with early onset Parkinson’s disease, as it demonstrates that pathophysiological phenotype was caused by the loss of enzymatic activity associated with the A107P mutation (Ghazavi et al., 2011).

Next, we focused on the putative DJ-1 oxyanion hole. The G74S mutation completely abolished activity, whereas a G75S mutation significantly reduced activity (Fig. 5, D and E). To rule out the possibility that the loss of activity by the DJ-1 mutants described above resulted from massive structural perturbations, we performed thermal shift analyses. Data confirmed that the protein structures of the G74S, G75S, C106S, and A107P mutants were maintained at room temperature (Tm = 68.3°C, 51.2°C, 56.2/63.0°C, 74.1°C, and 55.0°C for WT, G74S, G75S, C106S, and A107P, respectively) (Fig. 5, F and G).

To confirm the evolutionary importance of the nucleophilic cysteine, oxyanion hole, and hydrogen bond between the cPGA carbonyl group and the DJ-1 backbone nitrogen, we introduced equivalent mutations (C106S, A107P, G74S, and G75S) into YajL and analyzed their enzymatic effects. Activity in the C106S, A107P, and G74S mutants was completely abolished and was significantly reduced by the G75S mutation (Fig. 5, H and I), indicating the importance of the equivalent amino acids in YajL. These results support our hypothesis that the G74–G75 oxyanion hole, nucleophilic C106, and the neighboring A107 are directly involved in DJ-1 hydrolysis of cPGA.

### The P158 proximal to cPGA is essential for DJ-1 hydrolase activity

**Figure 6.**
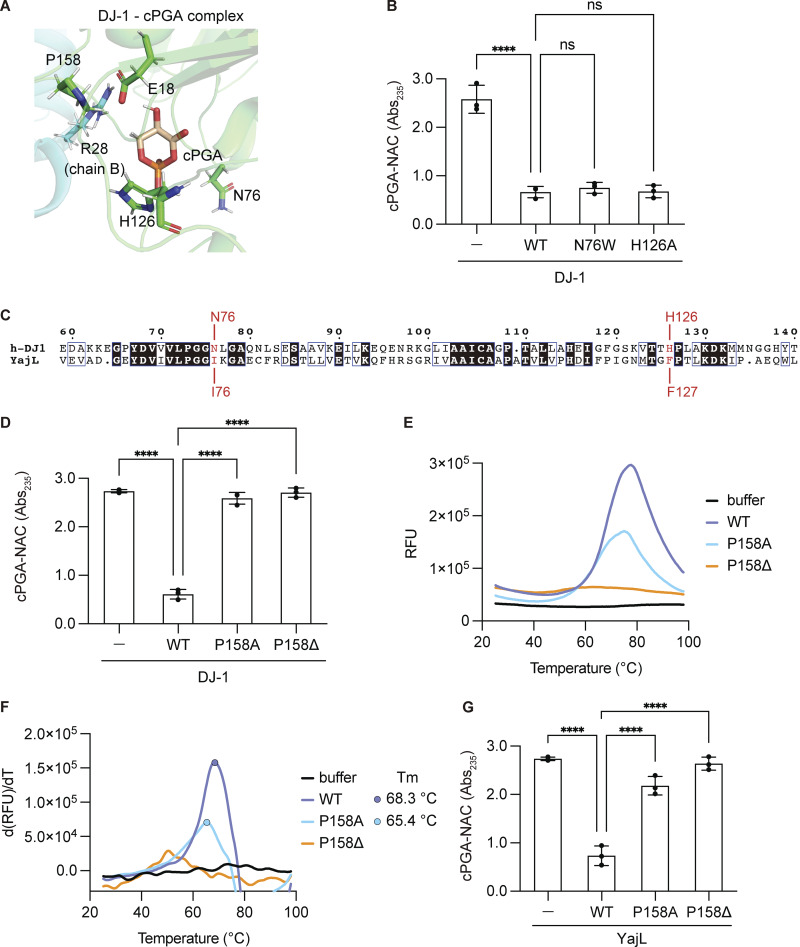
**P158 in DJ-1 is essential for cPGA hydrolase activity. (A)** The positional relationship of cPGA with N76, H126, and P158 in the DJ-1–cPGA molecular model. **(B)** The cPGA hydrolytic activity of the N76W and H126A mutants was monitored by a decrease in Abs_235_ (all *n* = 3). **(C)** Sequence alignment between human DJ-1 and *E. coli* YajL. DJ-1 N76 and H126 correspond to I76 and F127 in YajL (shown in red font). Identical and conserved amino acids are shown in black and blue boxes, respectively. **(D)** The cPGA hydrolytic activity of WT, P158A, or P158∆ DJ-1 was monitored by a decrease in Abs_235_ (all *n* = 3). **(E and F)** The folding stability of WT (blue), P158A (light blue), or P158∆ (orange) DJ-1 was measured using a protein thermal shift assay. Representative relative fluorescence unit (RFU) (E) and positive derivative [d(RFU)/dT] curves with melting temperatures (Tm) (F) are shown. The black line indicates the buffer control. **(G)** The cPGA hydrolytic activity of WT, P158A, and P158∆ YajL was monitored by a decrease in Abs_235_ (all *n* = 3). Data in B, D, and G are the mean ± SD of three experiments. *P < 0.05 using one-way ANOVA with Dunnett’s multiple comparisons test (B, D, and G).

To investigate the importance of other amino acids predicted to form the cPGA-binding pocket, we introduced mutations into N76, H126, and P158 (Fig. 6 A) and evaluated their enzymatic effects. Among these residues, H126 is especially intriguing, as it was suggested to be equivalent to the missing catalytic triad histidine that activates the nucleophilic cysteine (Tao and Tong, 2003). However, the H126A mutation, which removes the side chain, had no effect on activity (Fig. 6 B), refuting the hypothesis that H126 serves as the base equivalent to the conserved His in the catalytic triad. We also examined the importance of N76 by substituting with a bulkier amino acid (i.e., tryptophan; N76W in DJ-1) but found the change did not impair enzymatic activity (Fig. 6 B). These results indicate that despite the proximity of N76 and H126 to cPGA, these amino acids are not essential. The relatively low evolutionary conservation of these residues in YajL (N76 in DJ-1 versus I76 in YajL; H126 in DJ-1 versus F127 in YajL) also supports the conclusion (Fig. 6 C).

In contrast, we found that the P158A mutation completely abolished activity (Fig. 6 D), underscoring the critical role of this residue. Interestingly, P158 was identified as a pathogenic mutation site with P158Δ reported in a Dutch Parkinson’s disease patient whose age of onset was 34 (Macedo et al., 2009). This variant is a three-nucleotide deletion that results in loss of Pro158. We confirmed loss of enzymatic activity with the P158Δ mutation (Fig. 6 D), highlighting its physiological significance. Thermal shift data revealed that the P158A mutant maintains its structure, whereas the P158∆ mutation results in a misfolded DJ-1 protein (Fig. 6, E and F). Collectively, the catalytic and pathophysiological importance of P158 was revealed by the P158A and P158∆ mutations, although the latter mutation was not limited to enzymatic inactivation but also disruption of total protein structure. Equivalent mutations in YajL (P158A and P158∆) also abolished enzymatic activity (Fig. 6 G).

### Reaction mechanism for DJ-1 hydrolysis of cPGA

**Figure 7.**
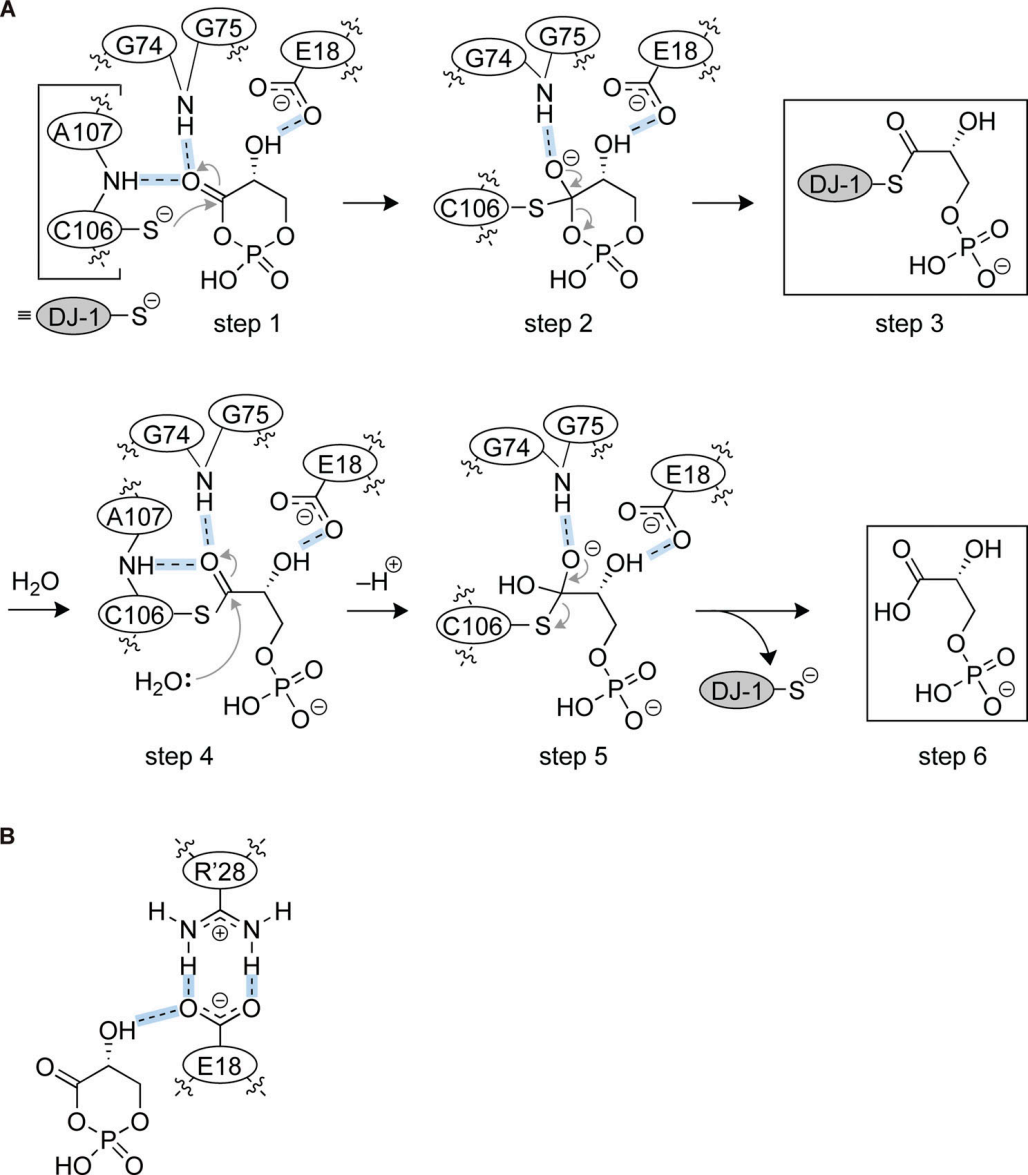
**Proposed reaction mechanism for DJ-1–mediated cPGA hydrolysis. (A)** The reaction is initiated following the formation of hydrogen bonds between the substrate (cPGA) and E18, G74–75, and A107 in the DJ-1 active site (step 1). Attack of C106 on the carbonyl carbon yields the first tetrahedral intermediate that is stabilized by the oxyanion hole comprised by G74 and G75 (step 2). Reformation of the carbon-oxygen double bond leads to formation of the acyl enzyme intermediate via a thioester linkage (step 3). Water then attacks the carbonyl carbon of the acyl enzyme (step 4) to form the second tetrahedral intermediate, which is similarly stabilized by the oxyanion hole as in step 2 (step 5). Lastly, reformation of the carbon-oxygen double bond restores the free enzyme and releases the 3PG product (step 6). **(B)** Schematic diagram showing how E18 interacts with cPGA. Hydrogen bond formation between E18 and the cPGA hydroxyl group is assisted by the intermolecular salt bridge formed with R28.

Although the physiological significance remains unclear, DJ-1 exhibits esterase activity in vitro, a function that we have analyzed in detail (Watanabe et al., 2024). Interestingly, α/β hydrolase fold esterases provide an important mechanistic insight to understanding DJ-1 hydrolysis of cPGA. The α/β hydrolase fold esterase enzymatic reaction proceeds via a ping-pong bi–bi mechanism involving an acyl enzyme intermediate (Rauwerdink and Kazlauskas, 2015). Namely, the R_1_-CO-O-R_2_ substrate interacts with the active site (step 1) of the enzyme, and then the catalytic Ser/Cys attacks the carbonyl carbon of the substrate to yield the first tetrahedral intermediate, T_d_1 (step 2). T_d_1 is then reformed to yield the acyl enzyme intermediate (step 3), and the substrate-derived alcohol (R_2_-OH) is released. In the next step, water attacks the carbonyl carbon of the acyl enzyme (step 4), and the second tetrahedral intermediate, T_d_2, is formed (step 5). Lastly, T_d_2 is reformed to release the second product (R_1_-COOH), and the enzyme is restored to the free state (step 6). Based on this reaction mechanism, we can propose a model of DJ-1 hydrolysis that accounts for observed mutational effects (Fig. 7 A). Because the C106 thiol localizes very close to the carbon atom of the cPGA carbonyl group and C106 has a depressed p*K*a of 5.4 that ensures C106 reactivity (Witt et al., 2008), it is reasonable that Cys106 attacks the cPGA carbonyl group (step 1 in Fig. 7 A) to form the tetrahedral intermediate (step 2 in Fig. 7 A). During these steps, we surmise that hydrogen bonds between cPGA and DJ-1 facilitate the reaction, as our structural model predicted that the backbone nitrogen of A107 and the carboxylate of E18 form hydrogen bonds with oxygen atoms on the carbonyl group and hydroxy groups of cPGA, respectively (step 1 in Fig. 7 A). Indeed, the A107P mutation, which blocks the backbone amine with the Pro side chain, resulted in the loss of enzyme activity, whereas the A107I mutation, which extended the side chain, or the A107H mutation, which added a bulkier side chain, had no effect on enzymatic activity (Fig. 5). Moreover, mutation of either E18 or R28, which determine the side chain arrangement of E18 (Fig. 7 B), also inhibited the enzymatic activity of DJ-1 (Fig. 4). These results support the accuracy of our model for describing the first reaction step. In the case of α/β hydrolase fold esterases, this transition state is stabilized by a hydrogen bond between the negative charge on the carbonyl group of the substrate and the main chain nitrogen of the sequential glycine residues (G146–G147), which form the oxyanion hole (Wogulis et al., 2006). Similarly, in the case of DJ-1, we infer that the oxyanion hole is formed by G74–G75. Indeed, mutations in G74 and G75 decreased cPGA hydrolase activity (Fig. 5). The tetrahedral intermediate then transitions to the acyl-enzyme intermediate. In the case of esterases and proteases, the initial cleaved product containing an alcohol or amino group is released. However, in the case of DJ-1, since the cPGA substrate is a cyclic molecule, the ring-opened substrate remains as the acyl-enzyme intermediate, resulting in the formation of an intermediate, in which C106 links with 3PG via a thioester bond (step 3 in Fig. 7 A). Finally, water attacks the thioester bond, and thus 3PG is released as the product, and the enzyme reaction is completed (steps 4–6 in Fig. 7 A). The molecular model of enzymatic reaction described here is well consistent with the results of our mutational analysis.

### Amino acid residues essential for recombinant DJ-1 to catalyze cPGA degradation are also critical for intracellular DJ-1–mediated reactions

**Figure 8.**
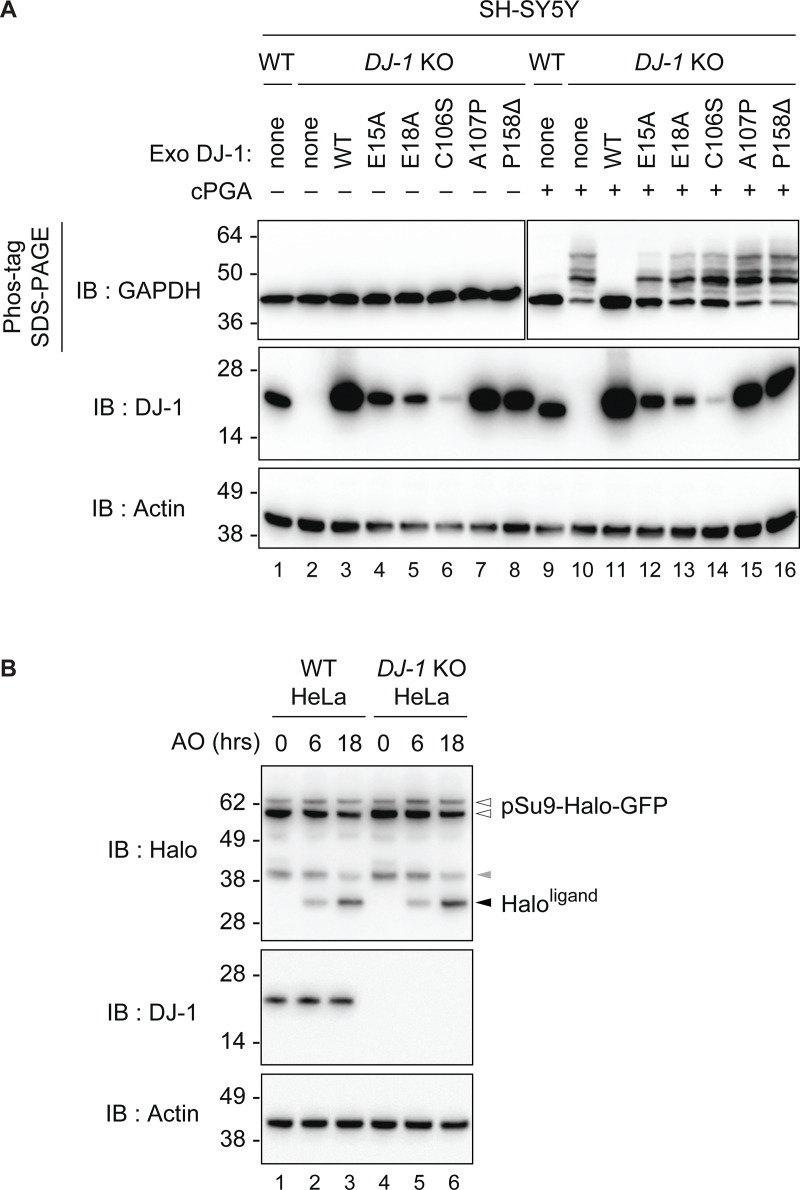
**Endogenous DJ-1 suppressed cPGA modification in cell lysates. (A)** Lysates of WT, *DJ-1* KO, or *DJ-1* KO SH-SY5Y cells expressing exogenous DJ-1 (WT or the indicated mutants) were treated with 1 mM cPGA and then phospho-glyceroyl modifications were detected by Phos-tag SDS-PAGE. The cells without exogenous DJ-1 expression are denoted as “none.” **(B)** WT or *DJ-1* KO HeLa cells stably expressing pSu9-Halo-mGFP and Parkin were pulse labeled with Halo ligand, followed by treatment with antimycin A and oligomycin (AO) for the indicated times, and then immunoblotted. A Halo^ligand^ band (black arrowhead) appeared in a time-dependent manner in both WT and *DJ-1* KO cells following AO treatment. The gray arrowhead indicates ∼40-kDa bands that are likely a cleavage product of the HaloTag construct generated during GFP chromophore formation. All blots are representative of at least two independent experiments. Source data are available for this figure: SourceData F8.

Although the results shown in Fig. 7 provided critical insights to understand the DJ-1–mediated cPGA hydrolysis reaction mechanism, we sought physiological confirmation of the mechanism by determining if the residues predicted critical for cPGA binding are essential for cPGA hydrolysis activity in cells. Because the thioester-mediated cPGA detection method (Fig. 1 A) is not suitable for cell lysates containing various impurities, we first had to establish a reliable method for detecting cPGA or cPGA-derived modifications. We focused on the ability of a 1,3-bis(bis(pyridin-2-ylmethyl)amino)propan-2-olato diMn(II) complex (referred to hereafter as Phos-tag) to capture phosphomonoester dianions (R-OPO_3_^2−^), which can facilitate acrylamide gel discrimination of phospho-serine/threonine proteins as slower migrating bands relative to their non-phosphorylated forms (Kinoshita et al., 2006). Because cPGA-modified proteins have N-phospho-glyceroyl-lysine with phosphomonoester dianions, we expected that the phos-tag system could be used to distinguish cPGA-modified proteins from intact ones. When cell lysates prepared from WT SH-SY5Y cells were incubated with 1 mM cPGA for 20 min and then subjected to PAGE-containing phos-tag, no mobility shifts in GAPDH were observed regardless of cPGA treatment (lanes 1 and 9 of Fig. 8 A). However, when lysates from *DJ-1* knockout (KO) cells were similarly assessed, a clear mobility shift in GAPDH was observed following cPGA treatment, suggesting that N-phospho-glyceroyl-modification of GAPDH occurs in the absence of a functional DJ-1 (lanes 2 and 10). The lack of a band shift in KO cells transfected with WT DJ-1 (lane 11) confirmed that the cPGA-derived phospho-glyceroyl modification is dependent on DJ-1 dysfunction. To investigate the role that residues identified in vitro (see Figs. 4, 5, and 6) are critical for DJ-1–mediated cPGA recognition and hydrolysis, we generated a series of mutants and exogenously expressed them in *DJ-1* KO cells. The E15A, E18A, C106S, A107P, and P158∆ mutations were unable to suppress the GAPDH mobility shift following cPGA treatment (Fig. 8 A, lanes 12–16), and their expression in the absence of cPGA had no effect on the GAPDH band shift (Fig. 8 A, lanes 4–8). Thus, the residues essential for recombinant DJ-1 recognition and degradation of cPGA are also indispensable for cellular DJ-1 function.

### DJ-1 is not essential for Parkin-mediated mitophagy

DJ-1 has also been reported to function as an essential downstream factor for Parkin (Imberechts et al., 2022). If so, Parkin-mediated mitophagy should be impaired when the cPGA hydrolysis activity of DJ-1 is lost. To examine this, we utilized a HaloTag processing assay (Yim et al., 2022) to monitor Parkin-mediated mitophagy following DJ-1 dysfunction. Halo is sensitive to lysosomal degradation but becomes resistant upon ligand binding. Consequently, it is possible to track autophagy using a Halo-tagged reporter protein followed by pulse labeling with the halo ligand. When delivered to lysosomes in response to autophagy, the reporter complex will be proteolytically processed to Halo^ligand^. This assay system has advantages such as the ability to measure the promotion of autophagy activity as a positive signal (an increase in Halo^ligand^ signal) and the use of the full-length Halo-tag reporter as an internal control. For this experiment, we used mitochondria-targeted Halo-GFP (pSu9-Halo-GFP) to monitor the mitophagy flux (Yim et al., 2022; Endo et al., 2024). WT or *DJ-1* KO HeLa cells stably expressing Parkin and pSu9-Halo-GFP were pulse labeled with the Halo ligand for 20 min and then treated with antimycin A and oligomycin to induce mitophagy. The Halo^ligand^ band appeared in both WT and *DJ-1* KO HeLa cells following antimycin A and oligomycin treatment in a time-dependent manner (Fig. 8 B, compare lanes 1–3 with 4–6), indicating that the mitophagy flux in HeLa cells was not inhibited by *DJ-1* deletion. As different results might be obtained in neurons, and DJ-1 dysfunction might promote Parkin-mediated mitophagy (Joselin et al., 2012), we cannot conclude that DJ-1’s cPGA hydrolase activity is unrelated to Parkin-mediated mitophagy. However, Fig. 8 B suggests that in HeLa cells, DJ-1 and its cPGA hydrolase activity are not essential for Parkin mitophagy.

### The physiologically significance of DJ-1–associated glyoxalase activity is limited

**Figure S1.**
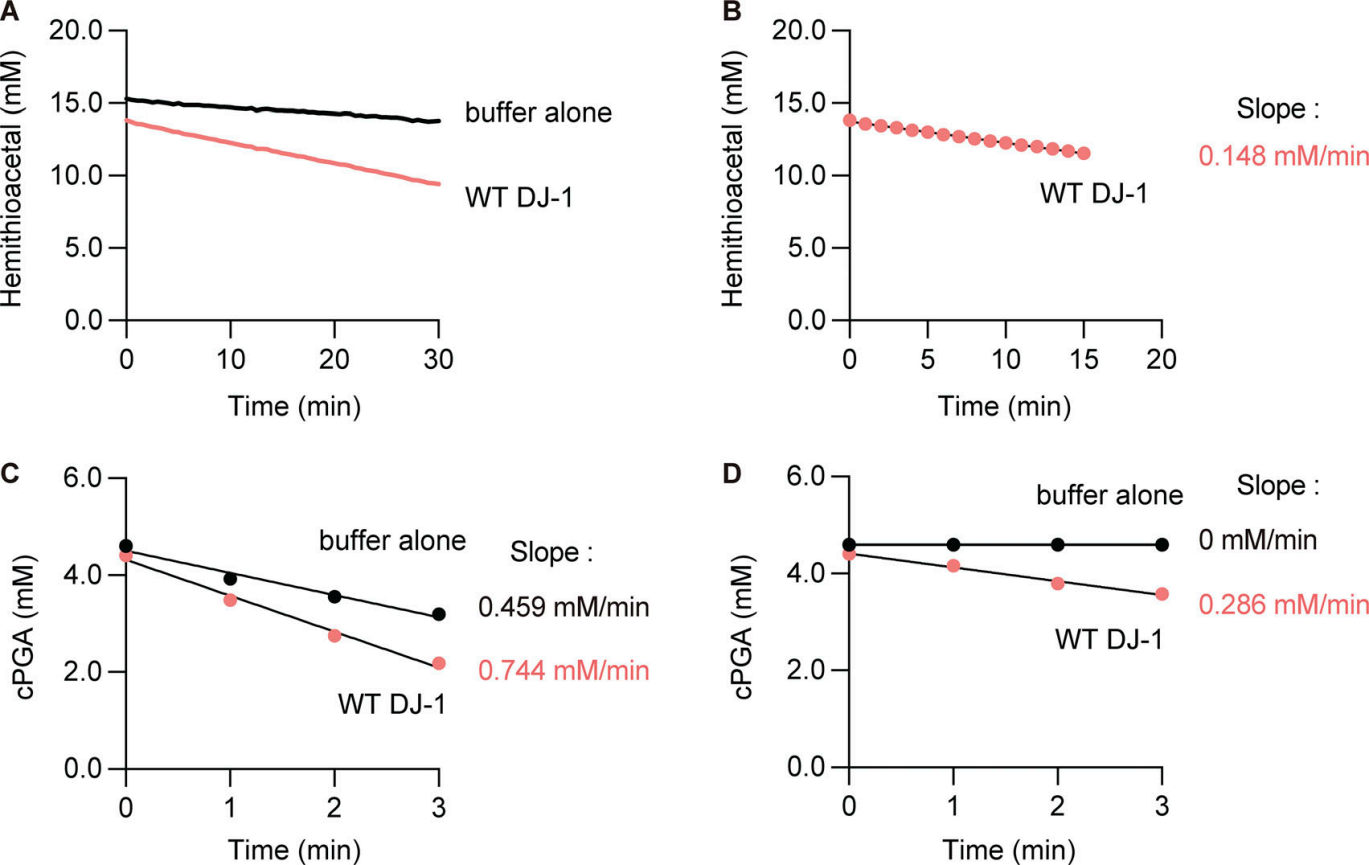
**Quantification of DJ-1 methylglyoxalase and cPGA hydrolase activities. (A and B)** Initial velocities of 15 mM MGO-derived hemithioacetal consumption when incubated with 10 µM DJ-1. A288 increases following hemithioacetal formation from MGO and N-acetyl-cysteine. *k*_cat_ was estimated as V_max_ divided by [E]_0_, and V_max_ for methylglyoxalase was determined by monitoring A288 changes during hemithioacetal turnover using various substrate concentrations and fitting initial velocities to a Michaelis–Menten plot. **(C and D)** Initial velocities of cPGA-derived hemithioacetal consumption when 4 mM cPGA was incubated with 10 nM DJ-1. For cPGA hydrolysis, substrate consumption was similarly monitored as A235 increases following hemithioacetal formation from cPGA and N-acetyl-cysteine. A235 changes was monitored under substrate-excess conditions, and initial velocity (V_0_) was calculated from the linear decrease in A235, corrected for spontaneous decay. *k*_cat_ was estimated as V_0_ divided by [E]_0_.

**Figure 9.**
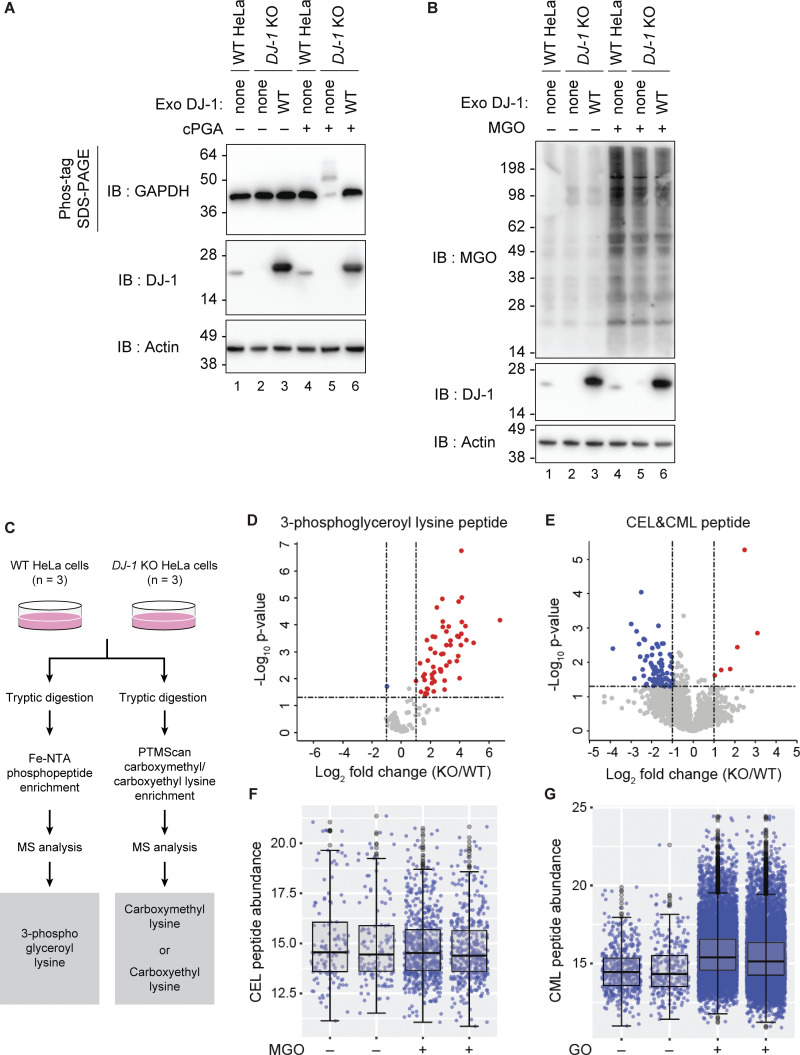
**Loss of endogenous DJ-1 had no effect on α-oxoaldehyde modifications but increased phospho-glyceroyl modifications. (A)** Cell lysates from WT, *DJ-1* KO, or *DJ-1* KO HeLa cells expressing exogenous WT DJ-1 were supplemented with cPGA. Phospho-glyceroyl modifications were analyzed by Phos-tag SDS-PAGE, followed by immunoblotting with an anti-GAPDH antibody. **(B)** The same lysates were treated with 2 mM MGO and immunoblotted with an anti-MGO antibody. The cells without exogenous DJ-1 expression are denoted as none. Blots are representative of at least two independent experiments (A and B). **(C)** Schematic overview of the experimental setup to assess the phosphoglycerate and α-oxoaldehyde modifications. **(D)** Volcano plot of the log_2_ fold change in 3-phosphoglyceroyl lysine peptide and the log_10_ of the P values (Student’s *t* test). Peptides with 3-phosphoglyceroyl lysine that significantly increased or decreased in *DJ-1* KO cells compared with WT cells (log_2_ fold change > 1 or log_2_ fold change less-than −1, P < 0.05) are shown in red (increase) and blue (decrease) circles, respectively. Mean fold changes and P values were calculated from three biological replicates (*n* = 3). **(E)** Volcano plot for CML and CEL peptides prepared as in D. **(F)** Abundance of CEL peptides in WT cells following treatment with MGO. Box plots show the distribution of values. Blue dots indicate individual data points, while gray dots represent outliers (*n* = 2). **(G)** Abundance of CML peptides in WT cells treated with glyoxal. Plots were drawn as in F (*n* = 2). Source data are available for this figure: SourceData F9.

In addition to cPGA hydrolase activity, DJ-1 has been reported to possess other enzymatic activities. Of these, the α-oxoaldehyde hydratase activity (glyoxalase III activity) of DJ-1 has been independently reported multiple times (Sun and Zheng, 2023; Wilson, 2011). We thus investigated which of the purported substrates (glyoxal, MGO, or cPGA) is most physiologically relevant. Among the activities associated with DJ-1, the enzymatic kinetics of cPGA hydrolysis are the highest. We previously determined that the DJ-1 glyoxalase activity *k*_cat_/*K*_m_ for phenylglyoxal (PGO) was 2.4 × 10^3^ s^−1^ M^−1^, whereas esterase activity with 4-nitrophenyl acetate was 1.2 × 10^3^ s^−1^ M^−1^ (Watanabe et al., 2024). In contrast, the reported *k*_cat_/*K*_m_ for cPGA hydrolytic activity was 5.9 × 10^6^ s^−1^ M^−1^ and thus was three orders of magnitude greater (Akhmadi et al., 2024). To gain further insights into the kinetics underlying DJ-1 utilization of MGO or cPGA as substrates, we performed rough estimations of the reactions and determined the methylglyoxalase *k*_cat_ was 0.38 ± 0.02 s^−1^, whereas the cPGA hydrolase was 420 ± 52 s^−1^ (Fig. S1). These values are almost consistent with a previous study (Akhmadi et al., 2024). The fact that the *k*_cat_ for cPGA is ∼1,000 times higher than for MGO suggests that cPGA hydrolysis is the genuine DJ-1 function. In addition, we used a cell-free assay system to determine if DJ-1 disruption also affects modifications derived from exogenous cPGA and MGO. When cPGA was added to lysates prepared from WT HeLa cells or *DJ-1* KO HeLa cells and analyzed by Phos-tag, a GAPDH mobility shift was observed exclusively in *DJ-1* KO cells (Fig. 9 A, lane 5), which is consistent with *DJ-1* KO in SH-SY5Y cells (Fig. 8). The GAPDH shift, however, disappeared when WT DJ-1 was reintroduced into *DJ-1* KO HeLa cells (lane 6). This confirmed that the loss of endogenous DJ-1 function accelerates phospho-glyceroylation of GAPDH. In contrast, when MGO was added to HeLa cell lysates and detected using an anti-MGO antibody, no differences in MGO modification were observed in any of the cells (WT, *DJ-1* KO, and *DJ-1* KO with exogenous DJ-1) assayed (Fig. 9 B, compare lanes 4–6). These results indicate that endogenous DJ-1 can clear exogenous cPGA but does not contribute to the clearance of exogenous MGO.

We next examined the role of DJ-1 dysfunction in a cell-based system on modifications derived from different endogenous sources, including glyoxal (i.e., carboxymethyl-lysine; CML), MGO (i.e., carboxyethyl-lysine; CEL), and cPGA (phospho-glyceroylation). Cell lysates from WT and *DJ-1* KO cells were digested with trypsin and then divided into two fractions. One fraction underwent phospho-peptide enrichment using Fe-NTA, while the other was processed with a PTMScan CML/CEL kit to enrich for peptides carrying CML or CEL. The enriched samples were then analyzed by mass spectrometry and compared (Fig. 9 C). As shown in the volcano plot in Fig. 9 D, peptides with cPGA-derived modifications (phospho-glyceroylation) were significantly increased in the *DJ-1* KO cells. Proteins identified in Fig. 9 D were listed in Table S1, and proteins involved in glycolysis (highlighted in yellow) and mitochondrial function (highlighted in green) were ranked among the top candidates. In contrast, DJ-1 dysfunction did not lead to a similar increase in peptides with the CML or CEL modifications (Fig. 9 E). To confirm that elevated MGO or glyoxal levels can increase CML and CEL modifications, WT cells were treated accordingly, and cell extracts were analyzed as before. An increase in both modification types was observed following MGO and glyoxal treatment (Fig. 9, F and G). Of the representative DJ-1 substrates identified so far, our data indicate that only cPGA fulfills the criteria to be considered a physiological substrate of endogenous DJ-1.

## Discussion

### Several issues remain in the known hydrolase/hydratase activities of DJ-1

Because various, and occasionally inconsistent, molecular functions have been ascribed to DJ-1, a consensus on its molecular function has yet to be reached. Nevertheless, the molecular structure of DJ-1 has provided useful clues to address this knowledge gap (Honbou et al., 2003; Huai et al., 2003; Lee et al., 2003; Tao and Tong, 2003; Wilson et al., 2003, 2005). Structural information revealed that the DJ-1 superfamily proteins (e.g., PfpI/Hsp31/DJ-1) have a conserved cysteine in a nucleophilic elbow structure that is reminiscent of cysteine proteases and α/β hydrolases. This strongly suggests that DJ-1 is a hydrolase and that its nucleophilic cysteine (C106) plays a critical enzymatic role. From this perspective, the molecular function of DJ-1 as a deglycase (Richarme et al., 2017), a cysteine protease (Koide-Yoshida et al., 2007; Olzmann et al., 2004), a glyoxalase (Lee et al., 2012; Richarme et al., 2015), an esterase (Vázquez-Mayorga et al., 2016; Watanabe et al., 2024), and a cPGA hydrolase (Akhmadi et al., 2024; Heremans et al., 2022) are intriguing as they all catalyze hydrolysis or hydration of their substrates. However, many of these proposed DJ-1 functions have scientific flaws.

The deglycase activity of DJ-1 is problematic because several reports implied that the activity is derived from the glyoxalase activity of DJ-1 acting on free MGO that is present in fast equilibrium with hemithioacetals and hemiaminals (Andreeva et al., 2019; Choi et al., 2023; Coukos et al., 2023; Gao et al., 2023; Mazza et al., 2022). The cysteine protease activity is controversial because the proteolytic activity reported has either been weak (Koide-Yoshida et al., 2007; Olzmann et al., 2004) or undetectable (Lee et al., 2003; Wilson et al., 2003). In addition, a rationale is needed to explain how DJ-1 can act as a protease despite the absence of the essential catalytic triad His (discussed in detail later).

Although DJ-1 can convert α-oxoaldehydes like MGO into α-hydroxy acids, such as lactate (Lee et al., 2012; Richarme et al., 2015) and glyoxalase, activity has been measured in vitro (Matsuda et al., 2017; Watanabe et al., 2024), and issues have been raised regarding the significance of this activity. Other glyoxal detoxification systems (e.g., the Glo1/2-mediated system) are more efficient than DJ-1. Indeed, the DJ-1 glyoxalase activity *k*_cat_ is ∼10^4^–10^5^ lower than the primary glutathione-dependent glyoxalase Glo1 (Mazza et al., 2022). Cellular studies further highlight this weakness as knockdown of Glo1 caused an accumulation of MGO adducts in cultured cells following MGO treatment, whereas no accumulation occurred following *DJ-1* KO (Heremans et al., 2022). We reconfirmed that DJ-1 dysfunction did not cause an accumulation of the MGO adduct (CEL) or the glyoxal adduct (CML) in cells (Fig. 9). Therefore, even though DJ-1 glyoxalase activity has been demonstrated in vitro, this activity appears to be secondary to the Glo1–2 system in cells, and thus its physiological relevance is questionable.

Like the glyoxalase activity, the physiological significance of DJ-1 esterase activity is still unclear. This activity is more pronounced than the glyoxalase activity in some respects. For example, we found that the esterase activity *k*_cat_ is 50 times higher than that of glyoxalase activity (Watanabe et al., 2024). However, because the esterase activity was monitored using an artificial substrate (4-nitrophenyl acetate), its physiological significance remains obscure. Moreover, this activity is not evolutionarily conserved. If the esterase activity is a genuine function of DJ-1, we can expect that YajL also possesses this activity. However, neither YajL nor the other *E. coli* DJ-1 homologs (HchA, YhbO, and ElbB) exhibited esterase activity (Watanabe et al., 2024).

While the molecular mechanism for cPGA hydrolase activity is undetermined, its activity was inferred from observations that phosphoglycerate-modified metabolites, presumably derived from cPGA, accumulated in DJ-1 KO cells (Heremans et al., 2022). However, due to the extreme instability of cPGA, no biochemical analysis had been conducted prior to 2024 (Akhmadi et al., 2024), and thus the molecular details were unclear.

An even more fundamental deficiency for the proposed DJ-1 activities is the lack of a mechanistic rationale accounting for the absence of a catalytic triad in DJ-1. Canonical Cys proteases and α/β hydrolases use the Ser/Cys-His-Asp catalytic triad to hydrolyze an acyl group-containing substrate, such as an ester or amide. Structural analysis, however, revealed that DJ-1 lacks this catalytic triad (Ser/Cys-His-Asp), which is essential for hydrolase/hydratase activity. Although Mazza et al. (2022) tried to explain the methylglyoxalase activity without relying on a catalytic triad (Mazza et al., 2022), no other models that logically explain how DJ-1 enzymatic activity proceeds without the catalytic triad have been proposed. Consequently, a reliable molecular mechanism that accounts for the atypical DJ-1 active site is needed. Given this background, we combined molecular modeling and biochemical analyses to elucidate the molecular mechanisms underlying the cPGA hydrolase activity of DJ-1.

### Reaction mechanism for DJ-1 hydrolysis of cPGA

In this study, we proposed a biochemically supported reaction mechanism for DJ-1 hydrolysis of cPGA based on the catalytic activity of α/β hydrolase fold esterases, which utilize a ping-pong bi–bi reaction mechanism. The process includes substrate binding (step 1 in Fig. 7 A), attack by catalytic Cys106 to form a tetrahedral intermediate via stabilization by an oxyanion hole (step 2), and formation of an acyl-enzyme intermediate (step 3). Unlike typical esterases, DJ-1 retains the ring-opened substrate as an intermediate due to the cyclic nature of cPGA. Water attacks the thioester bond (step 4) to form a tetrahedral intermediate (step 5), and DJ-1 finally releases 3PG as the product, and the enzyme is restored to its free state (step 6 in Fig. 7 A). To confirm the soundness of this proposed mechanism, we performed mutational analyses of key residues (e.g., E15, E18, A107, and P158) in vitro using recombinant DJ-1 (Figs. 4, 5, and 6) and with cell lysates incorporating cellular DJ-1 (Fig. 8). We speculated that the Pro side chain of the A107P mutation would block the backbone amine and thus prevent the formation of hydrogen bonds to the oxygen atoms on the cPGA carbonyl group that facilitate DJ-1 nucleophilic attack of cPGA (Fig. 7 A). Consistent with this hypothesis, DJ-1 enzymatic activity was lost in vitro and in cells when the A107P mutation occurred (Figs. 5 and 8). Similarly, impaired DJ-1 activity was observed following the introduction of mutations to E18 (Figs. 4 and 8), which our model predicts hydrogen bonds with the hydroxy group of cPGA that underpins the positioning of cPGA in the binding pocket (Fig. 7 A). Essential residues in DJ-1 and their proposed contributions to cPGA hydrolysis are summarized in Table 1.

**Table 1.** Essential residues in DJ-1 and their proposed contribution to the reaction mechanism

Essential residues in DJ-1	Proposed contribution to the cPGA hydrolysis
E15	Formation of the cPGA-binding pocket
E18	Formation of a hydrogen bond with the hydroxy group of cPGA
R28 (protomer)	Determination of the side chain arrangement of E18
G74	Stabilization of tetrahedral intermediate by oxyanion hole formation
G75	Stabilization of tetrahedral intermediate by oxyanion hole formation
C106	Nucleophilic cysteine that attacks the cPGA carbonyl group to form the tetrahedral intermediate
A107 (backbone nitrogen)	Formation of a hydrogen bond with oxygen atoms on the carbonyl group of cPGA
P158	Formation of the cPGA-binding pocket

### Physiologic relevance of cPGA hydrolase activity of DJ-1

In addition to cPGA hydrolase activity, DJ-1 has also been reported to exhibit α-oxoaldehyde hydratase activity, which converts glyoxal, MGO, and PGO into the corresponding α-hydroxy acids through hydration. Because these varied enzymatic activities are catalyzed by nucleophilic attack of DJ-1 C106 on the carbonyl groups of the respective substrates, they all proceed via a similar reaction mechanism. To assess the physiological relevance of the two reported DJ-1 enzymatic functions, it was necessary to compare the respective activities in WT cells and *DJ-1* KO cells. We thus established a novel experimental system using Phos-tag to monitor cPGA hydrolysis by endogenous DJ-1 in cell lysates. We found that the accumulation of cPGA modifications in the cell-free assay was inhibited by endogenous DJ-1, whereas the accumulation of α-oxoaldehyde–derived modifications was not (Fig. 9, A and B). Furthermore, mass spectrometry analysis of samples prepared from untreated (reagent-free) cells showed that cPGA modification accumulated in DJ-1–deficient cells, whereas MGO-derived and glyoxal-derived modifications did not (Fig. 9, C–G). These findings suggest that among the reported substrates, cPGA is most likely the physiological target of DJ-1.

### Two pathogenic missense mutations in DJ-1 abolish cPGA hydrolase activity

DJ-1 was identified as the causative gene for hereditary recessive Parkinson’s disease (PARK7) (Bonifati et al., 2003), and several pathogenic deletions/missense mutations associated with early onset of the disease have been identified (Corti et al., 2011; Repici and Giorgini, 2019; Trempe and Fon, 2013). However, to date, no report has examined if cPGA hydrolase activity is affected by the pathogenic mutations. Among the amino acids that form the cPGA hydrolase catalytic core in DJ-1 (Figs. 4, 5, and 6), A107 and P158 have been identified as pathogenic mutation sites—P158∆ in a Dutch patient and A107P in an Iranian patient (Ghazavi et al., 2011; Macedo et al., 2009). In our assay, we found that purified proteins harboring the respective mutations were devoid of cPGA hydrolase activity both in vitro (Figs. 5 and 6) and in cells, in which the mutations were introduced to DJ-1 (Fig. 8). Identification of pathogenic mutations that inhibited cPGA hydrolase activity strongly suggests a potential link between the early onset of Parkinson’s disease and increases in the reactive carbonyl compound cPGA.

### Conclusion

We integrated structural information obtained from models of DJ-1 complexed with cPGA and biochemical assays to gain a comprehensive understanding of the DJ-1 reaction mechanism. Our model used molecular simulations to predict potential interaction sites and biochemical data to assess the accuracy of the predictions. For example, the A107P mutation abolished enzymatic activity, whereas A107H and A107I mutations did not, suggesting that the hydrogen bond formed between the backbone amine of A107 and the cPGA carbonyl group is important for catalysis. Based on these findings, we proposed a credible molecular reaction model for cPGA hydrolytic activity in DJ-1 that provides a mechanistic rationale for how enzyme activity can proceed despite the absence of the traditional catalytic-triad motif (Fig. 7). Moreover, we revealed that two pathogenic missense mutations A107P and P158∆ abolished cPGA hydrolase activity (Figs. 5 and 6), suggesting the pathophysiological significance of the enzyme. Importantly, the mutant DJ-1 proteins lost cPGA hydrolytic activity in both the in vitro reconstituted system as well as cell lysates (Fig. 8). Moreover, in contrast to the glyoxal detoxification activity (i.e., an accepted enzymatic function of DJ-1), which remained unaffected in DJ-1–deficient cells, cPGA degradation activity was significantly reduced in cells lacking endogenous DJ-1 (Fig. 9). We believe that the molecular model presented here will provide solid insights for future functional studies of DJ-1 and enhance our understanding of the pathogenesis that leads to hereditary Parkinson’s disease.

## Materials and methods

### Plasmids

Plasmids for N-terminal His-tagged DJ-1 and C-terminal His-tagged HchA, YajL, YhbO, and ElbB expression were constructed by cloning the relevant genes into pET28a or pET21a vector as previously reported (Matsuda et al., 2017; Watanabe et al., 2024). Plasmids to express various DJ-1 and YajL mutants were generated using a classical two-step PCR method. The mutations were confirmed by Sanger DNA sequencing. Sequences for all of primers used in this study are listed in Table S2.

### Cells

HeLa (RRID:CVCL_0030), SH-SY5Y (RRID:CVCL_0019), and HEK293T (RRID:CVCL_0063) cells were cultured at 37°C with 5% CO_2_ in DMEM containing 1× nonessential amino acids, 1× sodium pyruvate, 1× penicillin–streptomycin–glutamine, and 10% fetal bovine serum. Cell lines used in this study were authenticated and tested for mycoplasma contamination. Preparation of HeLa cells stably expressing GFP-Parkin was previously reported (Okatsu et al., 2012). HeLa cells stably expressing Su9-Halo-mGFP were established by recombinant retrovirus infection. Virus particles were produced in HEK293T cells by co-transfection with Gag-Pol, VSV-G (RRID:Addgene_164440), and the retrovirus plasmid pMRX-IBU-pSu9-HaloTag7-mGFP (Yim et al., 2022) using Lipofectamine LTX Reagent (Invitrogen). After 12  h, the transfection medium was replaced with fresh medium, and the cells were further cultivated for 24 h. Collected viral supernatants were then added to HeLa cells with 8 µg/ml polybrene. *DJ-1* KO HeLa or SH-SY5Y cells transiently expressing DJ-1 mutants were established by transient transfection using FugeneHD Reagent (Promega) according to the manufacturer’s protocol.

### Purification of recombinant proteins

Recombinant proteins expressed from the DJ-1, HchA, YajL, YhbO, and ElbB plasmids described above were purified as previously reported (Matsuda et al., 2017; Watanabe et al., 2024). BL21(DE3)+RIL *E. coli* (Agilent Technologies) bacteria were transformed with each plasmid and cultured. The resultant pre-cultures (overnight culture of 2 ml LB media containing 25 μg/ml kanamycin or 100 μg/ml ampicillin) were subsequently added to 100 ml LB and cultured for 2 h at 32°C. Once the cultures reached an optical density of 0.3–0.5 at 600 nm, 0.3 mM IPTG was added, and the cultures were incubated for another 3 h at 32°C. Bacteria were collected by centrifugation at 5,800 *g* for 10 min. Pellets were resuspended in lysis buffer (20 mM Tris-buffer [pH 7.5], 200 mM NaCl, 10 mM *β*-mercaptoethanol, 1 µg/ml lysozyme [Wako], 1 µg/ml DNase I [Worthington], and 5 mM MgCl_2_) and sheared using a sonicator. Cellular debris was removed by centrifugation at 5,800 *g* for 10 min at 4°C, and the resulting supernatant was recovered. His-tagged recombinant proteins were purified using standard procedures with nickel-agarose (Ni-NTA Agarose, Qiagen) and relevant elution buffers (200 mM NaCl, 10 mM *β*-mercaptoethanol, and 250–500 mM imidazole in 20 mM Tris-buffer, pH 7.5). To remove the imidazole, the eluted samples were dialyzed twice against 1 liter of dialysis buffer (200 mM NaCl and 1 mM DTT in 20 mM Tris-buffer pH 7.5) for at least 4 h using 10K molecular weight cut-off cassettes (Thermo Fisher Scientific). Protein purity was confirmed by Coomassie brilliant blue–stained SDS-PAGE gels. Protein concentrations were determined using a BCA protein assay kit (Pierce).

### Measurement of phenylglyoxalase activity

To assess phenylglyoxalase activity, 2 µM purified protein (HchA and YhbO) was incubated with 1 mM PGO (TCI Chemicals) in 20 mM sodium phosphate buffer (pH 7.0) at 37°C for defined periods of time (Watanabe et al., 2024). PGO consumption was monitored by a reduction in Abs_250_ over time using an Enspire plate reader (PerkinElmer).

### Measurement of cPGA hydrolase activity

cPGA hydrolase activity was monitored via Abs_235_ based on the thioester generated when cPGA is reacted with NAC as described previously (Akhmadi et al., 2024). Fresh 50 mM cPGA was prepared by reacting 50 mM 3PG (SantaCruz) with 50 mM 1-ethyl-3-(3-dimethylaminopropyl)carbodiimide (TCI Chemicals) in 50 mM HCl on ice for 5 min. Synthesized cPGA (maximum concentration of 50 mM) was diluted to 1 mM, then quickly incubated with 1 µM recombinant protein (WT HchA, YhbO, and ElbB, or WT and various mutants of DJ-1 and YajL), 2 mM N-acetyl-L-cysteine (Sigma-Aldrich), and 1.8 mM NaOH in 50 mM sodium phosphate buffer (pH 7.0) at room temperature. After an initial 3-min incubation, Abs_235_ was monitored using a spectrophotometer (DeNovix).

### Computational modeling of DJ-1 and YajL

The crystal structure of DJ-1 in complex with a covalent inhibitor, 1-ethylindole-2,3-dione (PDB ID: 6AFI), (Tashiro et al., 2018) was used to model DJ-1 complexed with cPGA. Since DJ-1 functions as a homodimer in solution (Wilson et al., 2003), the dimeric model was used for our computational analysis. A cPGA model was initially built in GaussView 6 and was then fully optimized at the B3LYP/6-31G+(d,p) level with partial charges obtained by restrained electrostatic potential using HF/6-31G(d) single-point calculations on the optimized geometry using Gaussian 16 Rev C.02 (Frisch et al., 2016). The ANTECHAMBER module was used to parameterize the cPGA model (Wang et al., 2006).

The optimized cPGA model was docked into the dimeric DJ-1 crystal structure by superimposing the two hydroxy groups of cPGA onto the keto groups of 1-ethylindole-2,3-dione. The complex model was fully solvated with the TIP3P water model (Jorgensen et al., 1983) and 65 Na^+^ ions within a cubic periodic box possessing an edge length of 90 Å. The system was then neutralized by the addition of Cl^–^ counter ions using the AMBER LEaP module. The ff14SB force field (Maier et al., 2015) and the general AMBER force field 2 (Mobley et al., 2009) were employed for the protein and the substrate, respectively. Van der Waals interactions were truncated at a cutoff of 10 Å. The particle mesh Ewald method (Darden et al., 1993) was used to calculate electrostatic interactions. Initial relaxation of the system was accomplished through 200 steps of steepest descent minimization, with position restraints of 1,000 kcal mol^–1^ Å^–2^ imposed on the heavy atoms of the complex. The restraints were subsequently removed, and the entire system was subjected to 200 steps of steepest descent minimization. Next, to gradually heat the system, MD simulations were conducted over 1 ns at a temperature of 300 K using the *NPT* ensemble. The SHAKE algorithm (Ryckaert et al., 1977) was used to constrain bonds involving hydrogen atoms during the equilibration. The integration time step was set to 2 fs. The Berendsen weak coupling algorithm (Berendsen et al., 1984) was used to maintain a constant temperature and pressure. To further stabilize the DJ-1–cPGA complex, a 30-ns simulation was performed with several distance constraints (Table S3) enabled to maintain hydrogen bonds between the protein and ligand. We subsequently performed a 50-ns simulation in which the restraints were removed. The distance analysis for the unrestrained simulations was performed using the CPPTRAJ module (Roe and Cheatham, 2013). All energy minimization, equilibration, and production runs were performed using the PMEMD module of AMBER 22 (Case et al., 2023). The same procedure was used to build YajL–, ElbB–, HchA–, and YhbO–cPGA complex models, except that the initial coordinates were defined based on dimeric *E. coli* YajL (PDB ID: 2AB0) (Wilson et al., 2005), ElbB (PDB ID: 1VHQ) (Badger et al., 2005), HchA (PDB ID: 1IZY) (Lee et al., 2003), or YhbO (PDB ID: 1OI4) (Jung et al., 2012).

### Protein thermal shift assay

To monitor the thermal stability of recombinant proteins, WT and mutant proteins of ElbB, DJ-1, and YajL (30 µM) were incubated with Thermal Shift buffer and Protein Thermal Shift Dye provided in the manufacturer’s kit (Thermo Fisher Scientific). Fluorescence intensity was measured using the ROX reporter from a StepOnePlus Real-Time PCR system, with a ramp rate of 0.05°C/s per step (25°C–99°C). The melting temperature was determined using Protein Thermal Shift Software (Thermo Fisher Scientific).

### Preparation of *DJ-1* KO SH-SY5Y cells using CRISPR/Cas9 gene editing

*DJ-1* KO SH-SY5Y cell lines were established via CRISPR/Cas9-based genome editing. The gRNA target sequence (5′-TAA​GGT​CAC​CGT​TGC​AGG​CC-3′) for *DJ-1* exon three was designed using SYNTHEGO (https://design.synthego.com/#/). The oligonucleotide pairs were annealed and introduced into the BpiI site of the PX459 vector to yield PX459-DJ1-ex3. The resultant plasmid was transfected into SH-SY5Y cells. Puromycin-resistant cells were seeded into 96 well plates, and single clones were analyzed by immunoblotting to confirm *DJ-1* KO. Preparation of *DJ-1* KO HeLa cells was previously reported (Kojima et al., 2016).

### Conventional SDS-PAGE and Phos-tag SDS-PAGE

Cells grown in 6-well plates were washed twice with PBS and solubilized with 2% CHAPS buffer (25 mM HEPES-KOH, pH 7.5, 300 mM NaCl, 2% [wt/vol] CHAPS, and complete) on ice for 5 min. After centrifugation at 12,000 *g* for 3 min at 4°C, the supernatants were collected, and protein concentrations were determined by a spectrophotometer. SDS-PAGE sample buffer with DTT was added to the supernatants, followed by incubation at 95°C for 5 min, and the cell lysates were loaded onto 4–12% NuPAGE Bis-Tris gels (Thermo Fisher Scientific).

To detect phosphorylated proteins via PAGE, 10% polyacrylamide gels (10% acrylamide/bis Solution, 375 mM Tris-HCl, 0.1% SDS, 0.14% ammonium peroxodisulfate, and 0.1% N,N,N′,N′-tetramethylethylenediamine) containing 50 μM Phos-tag acrylamide (NARD Institute) and 100 μM MnCl_2_ were used. After electrophoresis, phos-tag acrylamide gels were washed with transfer buffer containing 0.01% SDS and 1 mM EDTA for 10 min with gentle shaking and then replaced with transfer buffer containing 0.01% SDS without EDTA for 10 min. Proteins were transferred to polyvinylidene difluoride (Millipore) membranes and analyzed by conventional immunoblotting.

### Immunoblotting

Proteins subjected to SDS-PAGE were transferred to polyvinylidene difluoride membranes and blocked with 1% (wt/vol) skim-milk/TBS-T for 30 min. To detect the indicated proteins, mouse anti–DJ-1 3E8 (ADI-KAM-SA100E; at 1:2,000 dilution; RRID:AB_2039445; ENZO), mouse anti-GAPDH (Cat #MAB374; 1:200; RRID:AB_2107445; Millipore), mouse anti-methylglyoxal (Cat #STA-011; 1:1,000; RRID:AB_3096122; Cell Biolabs), mouse anti-Halo (Cat #G9211; 1:1,000; RRID:AB_2688011; Promega), and mouse anti–Beta-actin (Cat #M177-3; 1:4,000; RRID:AB_10697039; MBL) antibodies were used as primary antibodies and incubated for 2 h. HRP-conjugated goat anti-mouse IgG (Cat #W4021; 1:10,000; RRID:AB_430834; Promega) was used as a secondary antibody and incubated for 45 min. Proteins were detected using a Western Lighting Plus-ECL Kit on a FUSION SOLO S system (VILBER).

### Cell-free cPGA hydrolase and methylglyoxalase assays

For the reconstitution of phosphoglyceroylation and methylglyoxylation in a cell-free assay, cell lysates were supplemented with cPGA or MGO and then immunoblotted. Specifically, cell lysates were prepared from WT HeLa cell, *DJ-1* KO HeLa cells, and *DJ-1* KO HeLa cells expressing exogenous DJ-1 using 2% CHAPS buffer (25 mM HEPES-KOH, pH 7.5, 300 mM NaCl, 2% [wt/vol] CHAPS, and protease inhibitor [cOmplete]) as described above. To obtain 25 mM cPGA, 25 mM 3PG and 25 mM 1-(3-dimethylaminopropyl)-3-ethylcarbodiimide hydrochloride were mixed with 25 mM HCl and incubated on ice for 5 min. Cell lysates were supplemented with 1 mM cPGA for 20 min, subjected to phos-tag PAGE, and immunoblotted using a mouse anti-GAPDH antibody (Cat #MAB374; 1:200; RRID:AB_2107445; Millipore) to monitor phosphoglyceroylation. To assess methylglyoxylation, a subset was incubated with 2 mM methylglyoxal (Sigma-Aldrich) at 37°C for 2 h and then immunoblotted with a mouse anti-methylglyoxal antibody (Cat #STA-011; 1:1,000; RRID:AB_3096122; Cell Biolabs).

### HaloTag processing assay

Cells stably expressing Parkin and pSu9-Halo-mGFP were pre-treated with 100 nM TMR-conjugated Halo ligand (Promega) for 20 min. After washing twice with PBS, the cells were cultivated in DMEM with 10 μM oligomycin (Selleck Chemicals), 4 μM antimycin A (Sigma-Aldrich), and 10 μM Q-VD (Selleck Chemicals) to induce mitophagy. The cells were incubated for 6 and 18 h, and then total cell lysates were prepared for immunoblotting.

### Quantification of enzymatic parameter (*k*_cat_)

To assess methylglyoxalase activity, 40 mM methylglyoxal (Sigma-Aldrich) and 40 mM N-acetyl-L-cysteine (Sigma-Aldrich) were preincubated at room temperature for 30 min in 50 mM sodium phosphate buffer (pH 7.0) to form a hemithioacetal. Five different concentrations of hemithioacetal (0–15 mM) were incubated with DJ-1 (10 µM), and changes in A288 over time were measured as previously reported (Matsuda et al., 2017; Watanabe et al., 2024). The initial velocities were obtained via linear regression of the resulting data and were fitted to a Michaelis–Menten plot using GraphPad Prism 10 (RRID:SCR_002798; GraphPad Software) to calculate V_max_. The *k*_cat_ of methylglyoxalase was calculated as V_max_ divided by [E]_0_ (10 µM).

Determination of the initial cPGA hydrolysis reaction velocities needed for Michaelis–Menten plots was hampered by the instability of cPGA and its damaging effect on DJ-1. We thus set up an experimental condition with excessive substrate relative to the enzyme, and followed substrate consumption over time, which was linear. In detail, 4 mM cPGA and 10 nM DJ-1 were mixed in 50 mM sodium phosphate buffer (pH 7.0) at room temperature, and the reaction mixture was sampled every 1 min, followed by incubation with 2 mM NAC neutralized by 8 mM NaOH. After further incubation for 2 min, 50 mM sodium phosphate buffer with 60 mM HCl was added to neutralize and terminate the reaction, at which point Abs235 was measured. Slopes of substrate consumption were determined from linear fits of reaction conditions with or without DJ-1. An initial velocity under this experimental condition (V_0_) was calculated from the decline in velocity of Abs235 after correction for the spontaneous decay of cPGA, and the *k*_cat_ was roughly estimated as V_0_ divided by [E]_0_ (10 nM). All measurements were repeated at least three times, and average values are shown.

### Enrichment of 3-phosphoglyceroyl lysine peptides and mass spectrometry

WT or *DJ-1* KO HeLa cells (*n* = 3) were lysed in 500 μl of 6 M guanidine-HCl, 100 mM HEPES-NaOH, pH 7.5, 10 mM TCEP, and 40 mM CAA. The lysates were dissolved by heating and sonication, followed by centrifugation at 20,000 g for 15 min at 4°C. The supernatants were recovered, and proteins (1 mg each) purified by methanol–chloroform precipitation were solubilized in 150 μl of 0.1% RapiGest (Waters) in 50 mM triethylammonium bicarbonate. After sonication, the protein solutions were digested overnight with 10 µg trypsin/Lys-C mix (Promega) at 37°C. The resulting peptide solutions were acidified with TFA, centrifuged, and used with a High-Select Fe-NTA phosphopeptide enrichment kit (Thermo Fisher Scientific). The eluates were acidified, desalted using GL-Tip SDB (GL Sciences), evaporated in a SpeedVac concentrator, and redissolved in 0.1% TFA and 3% acetonitrile. LC-MS/MS analysis of the resultant peptides was performed on a nanoElute 2 coupled with a timsTOF HT mass spectrometer (Bruker). The peptides were separated on a 75-μm inner diameter × 150-mm C18 reversed-phase column (Nikkyo Technos). The mobile phase consisted of 0.1% formic acid in water (solvent A) and 0.1% formic acid in acetonitrile (solvent B). Peptides were loaded onto the column at a flow rate of 0.2 μl/min starting at 3% B, which was linearly ramped to 32% B over 90 min, then raised to 95% B at 91 min, and held at that level until 101 min. The mass spectrometer was operated in parallel accumulation–serial fragmentation mode. The m/z range for both MS1 and MS2 spectra was 100–1,700, and the ion mobility range was 0.6–1.6 V·s/cm^3^. The ramp time was 100 ms, with a duty cycle of 100%. Each acquisition cycle consisted of 10 parallel accumulation–serial fragmentation MS2 scans. A polygon filter was applied to the m/z and ion mobility space to exclude low m/z, singly charged ions from precursor selection. The raw data were processed using FragPipe (v22.0). Database searches were performed with MSFragger (v4.1), employing the default parameters of the LFQ-phospho workflow against the UniProt (RRID:SCR_002380) human database (20,454 entries). Carbamidomethylation of cysteine (+57.0215 Da) was set as a fixed modification. The following variable modifications were included: acetylation of the protein N terminus (+42.0106 Da); oxidation of methionine (+15.9949 Da); phosphorylation (+79.9663 Da) of serine, threonine, or tyrosine; and 3-phosphoglyceroyl (+167.9824 Da) at lysine. The resulting identifications were filtered using Philosopher with default parameters (MS Booster was disabled), and IonQuant (v1.10.27) was used for quantification with default software settings. Intensities of peptides containing 3-phosphoglyceroyl lysine were imported onto Perseus (v2.1.3.0). Filtering of missing values was performed by removal of peptides that were not present in all samples. Finally, missing values were imputed using the Perseus function “replace missing values from normal distribution” with the parameters width, 0.3; down shift, 1.8; and mode, separate for each column.

### Enrichment of carboxymethyl/carboxyethyl lysine peptides and mass spectrometry

WT or *DJ-1* KO HeLa cells (*n* = 3, 1 mg each) were digested with trypsin/Lys-C mix as described above. The resulting peptide solutions were diluted sixfold with HBS (50 mM HEPES-NaOH, pH 7.5 and 150 mM NaCl), centrifuged, and used with a PTMScan Carboxymethyl/Carboxyethyl Lysine Motif kit (Cell Signaling Technology). The eluates in 0.15% TFA and 5% acetonitrile were desalted, evaporated, and redissolved in 0.1% TFA and 3% acetonitrile. LC-MS/MS analysis of the resultant peptides was performed as described above. Peptides were loaded onto the column at a flow rate of 0.2 μl/min, starting at 5% B and ramping linearly to 20% B by 40 min, then to 35% B by 60 min, followed by a rapid increase to 95% B at 61 min, where it was held until 65 min. Database searches were performed as described above, except that the following variable modifications were included: acetylation of the protein N terminus (+42.0106 Da); oxidation of methionine (+15.9949 Da); and carboxymethylation (+58.0055 Da) or carboxyethylation (+72.0211 Da) of lysine. Intensities of peptides containing carboxymethyl and carboxyethyl lysine were imported onto Perseus, and filtering and imputation of missing values were performed as described above.

In addition, WT HeLa cells (*n* = 2, 300 µg each) were treated with cell culture media containing 2 mM MGO or GO for 2 h and digested with 3 µg trypsin/Lys-C mix. After enrichment of carboxymethyl/carboxyethyl lysine peptides and mass spectrometry, the intensities of peptides containing carboxyethyl or carboxymethyl lysine were analyzed in R version 4.4.2 running in RStudio 2024.12.1 (RRID:SCR_000432).

### Statistical analysis

All data are presented as means ± SD, unless otherwise indicated. Statistical significance was determined using either one-way or two-way ANOVA with Bonferroni’s or Dunnett’s multiple comparison test. Data distribution was assumed to be normal, but this was not formally tested. Significance values are indicated as *P < 0.05. All statistical analyses were performed using Prism 10.

### Online supplemental material

Fig. S1 shows the quantification of DJ-1’s methylglyoxalase and cPGA hydrolase activities. Table S1 shows a list of proteins modified by 3-phosphoglyceroyl lysine in DJ-1 KO cells, related to Fig. 9 D. Table S2 contains the sequences for all of primers used in this study. Table S3 shows the distance restraints used for MD simulations of the DJ-1–cPGA model. Video 1 is a 50-ns MD simulation for DJ-1 in complex with cPGA.

## Supplementary Material

Table S1 shows a list of proteins modified by 3-phosphoglyceroyl lysine in DJ-1 KO cells, related to Fig. 9 D.

Table S2 contains the sequences for all of primers used in this study.

Table S3 shows the distance restraints used for MD simulations of the DJ-1–cPGA model.

SourceData F8 is the source file for Fig. 8.

SourceData F9 is the source file for Fig. 9.

## Data Availability

Original data obtained in this study are openly available in a public repository. The MS proteomics data have been deposited to the ProteomeXchange Consortium via the jPOST partner repository (https://repository.jpostdb.org/) with the dataset identifiers PXD063699, PXD063705, and PXD063706. The original absorbance data used to quantify cPGA underlying Figs. 1, 4, 5, 6, and S1, and the structure models predicted in silico underlying Figs. 2, 3, 5 A, and 6 A have been deposited in the Dryad database (https://datadryad.org/) and are openly available at https://doi.org/10.5061/dryad.3j9kd51wz.

## References

[bib1] Abdallah, J., M. Mihoub, V. Gautier, and G. Richarme. 2016. The DJ-1 superfamily members YhbO and YajL from Escherichia coli repair proteins from glycation by methylglyoxal and glyoxal. Biochem. Biophys. Res. Commun. 470:282–286. 10.1016/j.bbrc.2016.01.068 26774339

[bib2] Akhmadi, A., A. Yeskendir, N. Dey, A. Mussakhmetov, Z. Shatkenova, A. Kulyyassov, A. Andreeva, and D. Utepbergenov. 2024. DJ-1 protects proteins from acylation by catalyzing the hydrolysis of highly reactive cyclic 3-phosphoglyceric anhydride. Nat. Commun. 15:2004. 10.1038/s41467-024-46391-9 38443379 PMC10915168

[bib3] Andreeva, A., Z. Bekkhozhin, N. Omertassova, T. Baizhumanov, G. Yeltay, M. Akhmetali, D. Toibazar, and D. Utepbergenov. 2019. The apparent deglycase activity of DJ-1 results from the conversion of free methylglyoxal present in fast equilibrium with hemithioacetals and hemiaminals. J. Biol. Chem. 294:18863–18872. 10.1074/jbc.RA119.011237 31653696 PMC6901308

[bib4] Badger, J., J.M. Sauder, J.M. Adams, S. Antonysamy, K. Bain, M.G. Bergseid, S.G. Buchanan, M.D. Buchanan, Y. Batiyenko, J.A. Christopher, . 2005. Structural analysis of a set of proteins resulting from a bacterial genomics project. Proteins. 60:787–796. 10.1002/prot.20541 16021622

[bib5] Berendsen, H.J.C., J.P.M. Postma, W.F. Vangunsteren, A. Dinola, and J.R. Haak. 1984. Molecular-dynamics with coupling to an external bath. J. Chem. Phys. 81:3684–3690. 10.1063/1.448118

[bib6] Bonifati, V., P. Rizzu, M.J. van Baren, O. Schaap, G.J. Breedveld, E. Krieger, M.C. Dekker, F. Squitieri, P. Ibanez, M. Joosse, . 2003. Mutations in the DJ-1 gene associated with autosomal recessive early-onset parkinsonism. Science. 299:256–259. 10.1126/science.1077209 12446870

[bib7] Case, D.A., H.M. Aktulga, K. Belfon, D.S. Cerutti, G.A. Cisneros, V.W.D. Cruzeiro, N. Forouzesh, T.J. Giese, A.W. Götz, H. Gohlke, . 2023. AmberTools. J. Chem. Inf. Model. 63:6183–6191. 10.1021/acs.jcim.3c01153 37805934 PMC10598796

[bib8] Choi, D., J. Kim, S. Ha, K. Kwon, E.H. Kim, H.Y. Lee, K.S. Ryu, and C. Park. 2014. Stereospecific mechanism of DJ-1 glyoxalases inferred from their hemithioacetal-containing crystal structures. FEBS J. 281:5447–5462. 10.1111/febs.13085 25283443

[bib9] Choi, J., S. Tak, H.M. Jung, S. Cha, E. Hwang, D. Lee, J.H. Lee, K.S. Ryu, and C. Park. 2023. Kinetic evidence in favor of glyoxalase III and against deglycase activity of DJ-1. Protein Sci. 32:e4641. 10.1002/pro.4641 37060572 PMC10127264

[bib10] Corti, O., S. Lesage, and A. Brice. 2011. What genetics tells us about the causes and mechanisms of Parkinson’s disease. Physiol. Rev. 91:1161–1218. 10.1152/physrev.00022.2010 22013209

[bib11] Coukos, J.S., C.W. Lee, K.S. Pillai, H. Shah, and R.E. Moellering. 2023. PARK7 catalyzes stereospecific detoxification of methylglyoxal consistent with glyoxalase and not deglycase function. Biochemistry. 62:3126–3133. 10.1021/acs.biochem.3c00325 37884446 PMC10634309

[bib12] Darden, T., D. York, and L. Pedersen. 1993. Particle mesh ewald - an N.Log(N) method for ewald sums in large systems. J. Chem. Phys. 98:10089–10092. 10.1063/1.464397

[bib13] Dimitriou, P.S., A.I. Denesyuk, T. Nakayama, M.S. Johnson, and K. Denessiouk. 2019. Distinctive structural motifs co-ordinate the catalytic nucleophile and the residues of the oxyanion hole in the alpha/beta-hydrolase fold enzymes. Protein Sci. 28:344–364. 10.1002/pro.3527 30311984 PMC6319758

[bib14] Endo, R., H. Kinefuchi, M. Sawada, R. Kikuchi, W. Kojima, N. Matsuda, and K. Yamano. 2024. TBK1 adaptor AZI2/NAP1 regulates NDP52-driven mitochondrial autophagy. J. Biol. Chem. 300:107775. 10.1016/j.jbc.2024.107775 39276928 PMC11490886

[bib15] Frisch, M.J., G.W. Trucks, H.B. Schlegel, G.E. Scuseria, M.A. Robb, J.R. Cheeseman, G. Scalmani, V. Barone, G.A. Petersson, H. Nakatsuji, . 2016. Gaussian 16, Revision B.01. Gaussian, Inc., Wallingford, CT, USA.

[bib16] Gao, Q., J.W. Jacob-Dolan, and R.A. Scheck. 2023. Parkinsonism-associated protein DJ-1 is an antagonist, not an eraser, for protein glycation. Biochemistry. 62:1181–1190. 10.1021/acs.biochem.3c00028 36820886 PMC10035033

[bib17] Ghazavi, F., Z. Fazlali, S.S. Banihosseini, S.R. Hosseini, M.H. Kazemi, S. Shojaee, K. Parsa, H. Sadeghi, F. Sina, M. Rohani, . 2011. PRKN, DJ-1, and PINK1 screening identifies novel splice site mutation in PRKN and two novel DJ-1 mutations. Mov. Disord. 26:80–89. 10.1002/mds.23417 21322020

[bib18] Heremans, I.P., F. Caligiore, I. Gerin, M. Bury, M. Lutz, J. Graff, V. Stroobant, D. Vertommen, A.A. Teleman, E. Van Schaftingen, and G.T. Bommer. 2022. Parkinson’s disease protein PARK7 prevents metabolite and protein damage caused by a glycolytic metabolite. Proc. Natl. Acad. Sci. USA. 119:e2111338119. 10.1073/pnas.2111338119 35046029 PMC8795555

[bib19] Honbou, K., N.N. Suzuki, M. Horiuchi, T. Niki, T. Taira, H. Ariga, and F. Inagaki. 2003. The crystal structure of DJ-1, a protein related to male fertility and Parkinson’s disease. J. Biol. Chem. 278:31380–31384. 10.1074/jbc.M305878200 12796482

[bib20] Huai, Q., Y. Sun, H. Wang, L.S. Chin, L. Li, H. Robinson, and H. Ke. 2003. Crystal structure of DJ-1/RS and implication on familial Parkinson’s disease. FEBS Lett. 549:171–175. 10.1016/S0014-5793(03)00764-6 12914946

[bib21] Imberechts, D., I. Kinnart, F. Wauters, J. Terbeek, L. Manders, K. Wierda, K. Eggermont, R.F. Madeiro, C. Sue, C. Verfaillie, and W. Vandenberghe. 2022. DJ-1 is an essential downstream mediator in PINK1/parkin-dependent mitophagy. Brain. 145:4368–4384. 10.1093/brain/awac313 36039535 PMC9762950

[bib22] Jorgensen, W.L., J. Chandrasekhar, J.D. Madura, R.W. Impey, and M.L. Klein. 1983. Comparison of simple potential functions for simulating liquid water. J. Chem. Phys. 79:926–935. 10.1063/1.445869

[bib23] Joselin, A.P., S.J. Hewitt, S.M. Callaghan, R.H. Kim, Y.-H. Chung, T.W. Mak, J. Shen, R.S. Slack, and D.S. Park. 2012. ROS-dependent regulation of Parkin and DJ-1 localization during oxidative stress in neurons. Hum. Mol. Genet. 21:4888–4903. 10.1093/hmg/dds325 22872702

[bib24] Jung, H.J., S. Kim, Y.J. Kim, M.K. Kim, S.G. Kang, J.H. Lee, W. Kim, and S.S. Cha. 2012. Dissection of the dimerization modes in the DJ-1 superfamily. Mol. Cells. 33:163–171. 10.1007/S10059-012-2220-6 22228183 PMC3887719

[bib25] Kahle, P.J., J. Waak, and T. Gasser. 2009. DJ-1 and prevention of oxidative stress in Parkinson’s disease and other age-related disorders. Free Radic. Biol. Med. 47:1354–1361. 10.1016/j.freeradbiomed.2009.08.003 19686841

[bib26] Kinoshita, E., E. Kinoshita-Kikuta, K. Takiyama, and T. Koike. 2006. Phosphate-binding tag, a new tool to visualize phosphorylated proteins. Mol. Cell. Proteomics. 5:749–757. 10.1074/mcp.T500024-MCP200 16340016

[bib27] Koide-Yoshida, S., T. Niki, M. Ueda, S. Himeno, T. Taira, S.M. Iguchi-Ariga, Y. Ando, and H. Ariga. 2007. DJ-1 degrades transthyretin and an inactive form of DJ-1 is secreted in familial amyloidotic polyneuropathy. Int. J. Mol. Med. 19:885–893. 10.3892/ijmm.19.6.885 17487420

[bib28] Kojima, W., Y. Kujuro, K. Okatsu, Q. Bruno, F. Koyano, M. Kimura, K. Yamano, K. Tanaka, and N. Matsuda. 2016. Unexpected mitochondrial matrix localization of Parkinson’s disease-related DJ-1 mutants but not wild-type DJ-1. Genes Cells. 21:772–788. 10.1111/gtc.12382 27270837

[bib29] Lee, C., J. Lee, J.Y. Lee, and C. Park. 2016. Characterization of the Escherichia coli YajL, YhbO and ElbB glyoxalases. FEMS Microbiol. Lett. 363:fnv239. 10.1093/femsle/fnv239 26678554

[bib30] Lee, J.Y., J. Song, K. Kwon, S. Jang, C. Kim, K. Baek, J. Kim, and C. Park. 2012. Human DJ-1 and its homologs are novel glyoxalases. Hum. Mol. Genet. 21:3215–3225. 10.1093/hmg/dds155 22523093

[bib31] Lee, S.J., S.J. Kim, I.K. Kim, J. Ko, C.S. Jeong, G.H. Kim, C. Park, S.O. Kang, P.G. Suh, H.S. Lee, and S.S. Cha. 2003. Crystal structures of human DJ-1 and Escherichia coli Hsp31, which share an evolutionarily conserved domain. J. Biol. Chem. 278:44552–44559. 10.1074/jbc.M304517200 12939276

[bib32] Lo, M.C., A. Aulabaugh, G. Jin, R. Cowling, J. Bard, M. Malamas, and G. Ellestad. 2004. Evaluation of fluorescence-based thermal shift assays for hit identification in drug discovery. Anal. Biochem. 332:153–159. 10.1016/j.ab.2004.04.031 15301960

[bib33] Macedo, M.G., D. Verbaan, Y. Fang, S.M. van Rooden, M. Visser, B. Anar, A. Uras, J.L. Groen, P. Rizzu, J.J. van Hilten, and P. Heutink. 2009. Genotypic and phenotypic characteristics of Dutch patients with early onset Parkinson’s disease. Mov. Disord. 24:196–203. 10.1002/mds.22287 18973254

[bib34] Maier, J.A., C. Martinez, K. Kasavajhala, L. Wickstrom, K.E. Hauser, and C. Simmerling. 2015. ff14SB: Improving the accuracy of protein side chain and backbone parameters from ff99SB. J. Chem. Theory Comput. 11:3696–3713. 10.1021/acs.jctc.5b00255 26574453 PMC4821407

[bib35] Matsuda, N., M. Kimura, B.B. Queliconi, W. Kojima, M. Mishima, K. Takagi, F. Koyano, K. Yamano, T. Mizushima, Y. Ito, and K. Tanaka. 2017. Parkinson’s disease-related DJ-1 functions in thiol quality control against aldehyde attack in vitro. Sci. Rep. 7:12816. 10.1038/s41598-017-13146-0 28993701 PMC5634459

[bib36] Mazza, M.C., S.C. Shuck, J. Lin, M.A. Moxley, J. Termini, M.R. Cookson, and M.A. Wilson. 2022. DJ-1 is not a deglycase and makes a modest contribution to cellular defense against methylglyoxal damage in neurons. J. Neurochem. 162:245–261. 10.1111/jnc.15656 35713360 PMC9539984

[bib37] Mobley, D.L., C.I. Bayly, M.D. Cooper, M.R. Shirts, and K.A. Dill. 2009. Small molecule hydration free energies in explicit solvent: An extensive test of fixed-charge atomistic simulations. J. Chem. Theory Comput. 5:350–358. 10.1021/ct800409d 20150953 PMC2701304

[bib38] Moellering, R.E., and B.F. Cravatt. 2013. Functional lysine modification by an intrinsically reactive primary glycolytic metabolite. Science. 341:549–553. 10.1126/science.1238327 23908237 PMC4005992

[bib39] Nardini, M., and B.W. Dijkstra. 1999. Alpha/beta hydrolase fold enzymes: The family keeps growing. Curr. Opin. Struct. Biol. 9:732–737. 10.1016/S0959-440X(99)00037-8 10607665

[bib40] Okatsu, K., T. Oka, M. Iguchi, K. Imamura, H. Kosako, N. Tani, M. Kimura, E. Go, F. Koyano, M. Funayama, . 2012. PINK1 autophosphorylation upon membrane potential dissipation is essential for Parkin recruitment to damaged mitochondria. Nat. Commun. 3:1016. 10.1038/ncomms2016 22910362 PMC3432468

[bib41] Olzmann, J.A., K. Brown, K.D. Wilkinson, H.D. Rees, Q. Huai, H. Ke, A.I. Levey, L. Li, and L.S. Chin. 2004. Familial Parkinson’s disease-associated L166P mutation disrupts DJ-1 protein folding and function. J. Biol. Chem. 279:8506–8515. 10.1074/jbc.M311017200 14665635

[bib42] Prahlad, J., D.N. Hauser, N.M. Milkovic, M.R. Cookson, and M.A. Wilson. 2014. Use of cysteine-reactive cross-linkers to probe conformational flexibility of human DJ-1 demonstrates that Glu18 mutations are dimers. J. Neurochem. 130:839–853. 10.1111/jnc.12763 24832775 PMC4156530

[bib43] Queliconi, B.B., W. Kojima, M. Kimura, K. Imai, C. Udagawa, C. Motono, T. Hirokawa, S. Tashiro, J.M.M. Caaveiro, K. Tsumoto, . 2021. Unfolding is the driving force for mitochondrial import and degradation of the Parkinson’s disease-related protein DJ-1. J. Cell Sci. 134:jcs258653. 10.1242/jcs.258653 34676411 PMC8645234

[bib44] Rauwerdink, A., and R.J. Kazlauskas. 2015. How the same core catalytic machinery catalyzes 17 different reactions: The serine-histidine-aspartate catalytic triad of α/β-Hydrolase fold enzymes. ACS Catal. 5:6153–6176. 10.1021/acscatal.5b01539 28580193 PMC5455348

[bib45] Repici, M., and F. Giorgini. 2019. DJ-1 in Parkinson’s disease: Clinical insights and therapeutic perspectives. J. Clin. Med. 8:1377. 10.3390/jcm8091377 31484320 PMC6780414

[bib46] Richarme, G., C. Liu, M. Mihoub, J. Abdallah, T. Leger, N. Joly, J.C. Liebart, U.V. Jurkunas, M. Nadal, P. Bouloc, . 2017. Guanine glycation repair by DJ-1/Park7 and its bacterial homologs. Science. 357:208–211. 10.1126/science.aag1095 28596309

[bib47] Richarme, G., M. Mihoub, J. Dairou, L.C. Bui, T. Leger, and A. Lamouri. 2015. Parkinsonism-associated protein DJ-1/Park7 is a major protein deglycase that repairs methylglyoxal- and glyoxal-glycated cysteine, arginine, and lysine residues. J. Biol. Chem. 290:1885–1897. 10.1074/jbc.M114.597815 25416785 PMC4340429

[bib48] Roe, D.R., and T.E. Cheatham III. 2013. PTRAJ and CPPTRAJ: Software for processing and analysis of molecular dynamics trajectory data. J. Chem. Theory Comput. 9:3084–3095. 10.1021/ct400341p 26583988

[bib49] Ryckaert, J.P., G. Ciccotti, and H.J.C. Berendsen. 1977. Numerical-integration of cartesian equations of motion of a system with constraints - Molecular-dynamics of N-Alkanes. J. Comput. Phys. 23:327–341. 10.1016/0021-9991(77)90098-5

[bib50] Semisotnov, G.V., N.A. Rodionova, O.I. Razgulyaev, V.N. Uversky, A.F. Gripas’, and R.I. Gilmanshin. 1991. Study of the “molten globule” intermediate state in protein folding by a hydrophobic fluorescent probe. Biopolymers. 31:119–128. 10.1002/bip.360310111 2025683

[bib51] Subedi, K.P., D. Choi, I. Kim, B. Min, and C. Park. 2011. Hsp31 of Escherichia coli K-12 is glyoxalase III. Mol. Microbiol. 81:926–936. 10.1111/j.1365-2958.2011.07736.x 21696459

[bib52] Sun, M.E., and Q. Zheng. 2023. The tale of DJ-1 (PARK7): A Swiss army knife in biomedical and psychological research. Int. J. Mol. Sci. 24:7409. 10.3390/ijms24087409 37108572 PMC10138432

[bib53] Taira, T., Y. Saito, T. Niki, S.M. Iguchi-Ariga, K. Takahashi, and H. Ariga. 2004. DJ-1 has a role in antioxidative stress to prevent cell death. EMBO Rep. 5:213–218. 10.1038/sj.embor.7400074 14749723 PMC1298985

[bib54] Tao, X., and L. Tong. 2003. Crystal structure of human DJ-1, a protein associated with early onset Parkinson’s disease. J. Biol. Chem. 278:31372–31379. 10.1074/jbc.M304221200 12761214

[bib55] Tashiro, S., J.M.M. Caaveiro, M. Nakakido, A. Tanabe, S. Nagatoishi, Y. Tamura, N. Matsuda, D. Liu, Q.Q. Hoang, and K. Tsumoto. 2018. Discovery and optimization of inhibitors of the Parkinson’s disease associated protein DJ-1. ACS Chem. Biol. 13:2783–2793. 10.1021/acschembio.8b00701 30063823 PMC6370461

[bib56] Trempe, J.F., and E.A. Fon. 2013. Structure and function of Parkin, PINK1, and DJ-1, the three musketeers of neuroprotection. Front. Neurol. 4:38. 10.3389/fneur.2013.00038 23626584 PMC3630392

[bib57] Vázquez-Mayorga, E., A.G. Díaz-Sánchez, R.K. Dagda, C.A. Domínguez-Solís, R.Y. Dagda, C.K. Coronado-Ramírez, and A. Martínez-Martínez. 2016. Novel redox-dependent esterase activity (EC 3.1.1.2) for DJ-1: Implications for Parkinson’s disease. Int. J. Mol. Sci. 17:1346. 10.3390/ijms17081346 27556455 PMC5000742

[bib58] Wang, J., W. Wang, P.A. Kollman, and D.A. Case. 2006. Automatic atom type and bond type perception in molecular mechanical calculations. J. Mol. Graph. Model. 25:247–260. 10.1016/j.jmgm.2005.12.005 16458552

[bib59] Watanabe, A., F. Koyano, K. Imai, Y. Hizukuri, S. Ogiwara, T. Ito, J. Miyamoto, C. Shibuya, M. Kimura, K. Toriumi, . 2024. The origin of esterase activity of Parkinson’s disease causative factor DJ-1 implied by evolutionary trace analysis of its prokaryotic homolog HchA. J. Biol. Chem. 300:107476. 10.1016/j.jbc.2024.107476 38879013 PMC11301059

[bib60] Wilson, M.A. 2011. The role of cysteine oxidation in DJ-1 function and dysfunction. Antioxid. Redox Signal. 15:111–122. 10.1089/ars.2010.3481 20812780 PMC3110098

[bib61] Wilson, M.A., J.L. Collins, Y. Hod, D. Ringe, and G.A. Petsko. 2003. The 1.1-A resolution crystal structure of DJ-1, the protein mutated in autosomal recessive early onset Parkinson’s disease. Proc. Natl. Acad. Sci. USA. 100:9256–9261. 10.1073/pnas.1133288100 12855764 PMC170905

[bib62] Wilson, M.A., D. Ringe, and G.A. Petsko. 2005. The atomic resolution crystal structure of the YajL (ThiJ) protein from Escherichia coli: A close prokaryotic homologue of the parkinsonism-associated protein DJ-1. J. Mol. Biol. 353:678–691. 10.1016/j.jmb.2005.08.033 16181642

[bib63] Witt, A.C., M. Lakshminarasimhan, B.C. Remington, S. Hasim, E. Pozharski, and M.A. Wilson. 2008. Cysteine pKa depression by a protonated glutamic acid in human DJ-1. Biochemistry. 47:7430–7440. 10.1021/bi800282d 18570440 PMC2760839

[bib64] Wogulis, M., C.E. Wheelock, S.G. Kamita, A.C. Hinton, P.A. Whetstone, B.D. Hammock, and D.K. Wilson. 2006. Structural studies of a potent insect maturation inhibitor bound to the juvenile hormone esterase of Manduca sexta. Biochemistry. 45:4045–4057. 10.1021/bi0521644 16566578 PMC4275126

[bib65] Yim, W.W., H. Yamamoto, and N. Mizushima. 2022. A pulse-chasable reporter processing assay for mammalian autophagic flux with HaloTag. Elife. 11:e78923. 10.7554/eLife.78923 35938926 PMC9385206

