# Supplementary material for: The reaction mechanism for glycolysis side product degradation by Parkinson’s disease–linked DJ-1
Source: J Cell Biol. 2025 Jun 4;224(8):e202411078. doi: 10.1083/jcb.202411078 (PMC12135873; doi:10.1083/jcb.202411078)

Fig 9A Source data

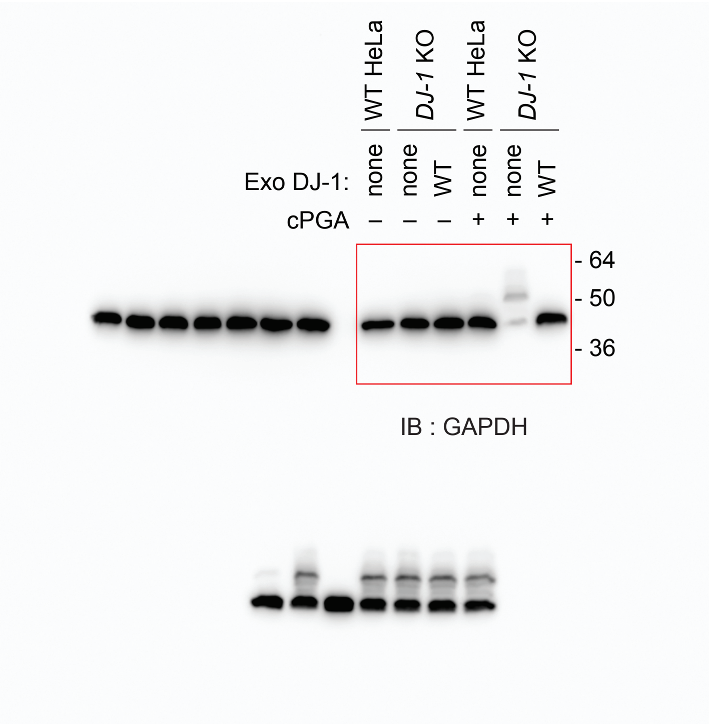

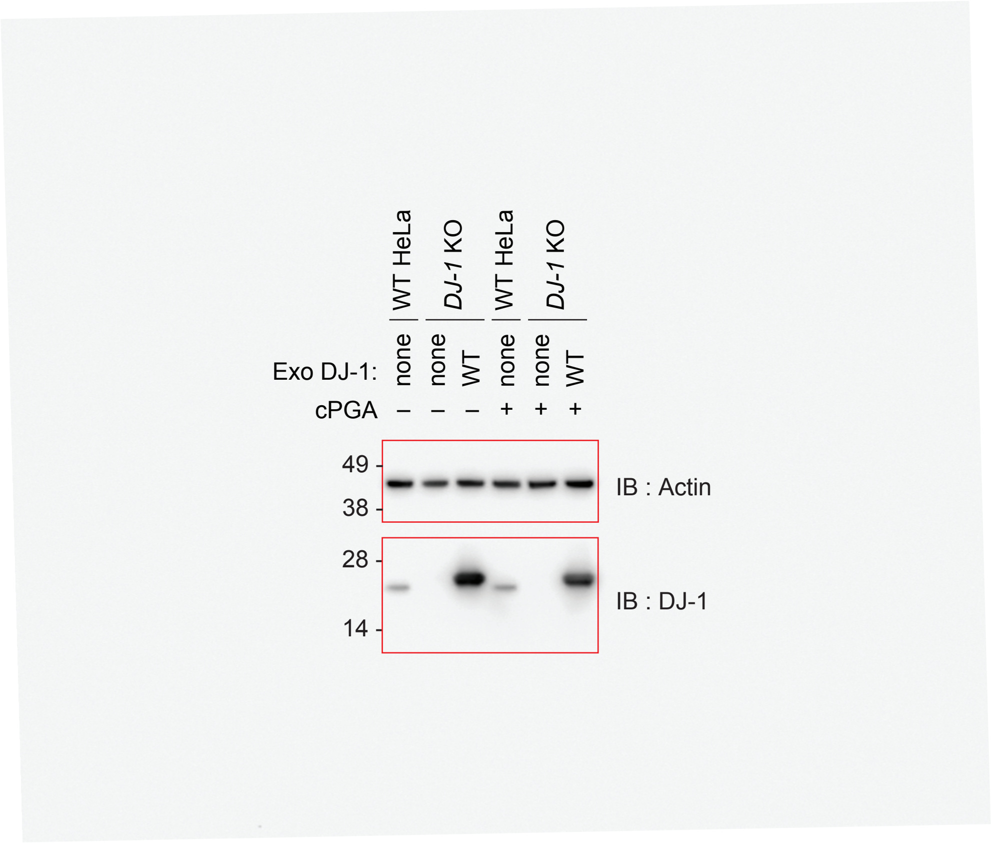

Fig 9B Source data

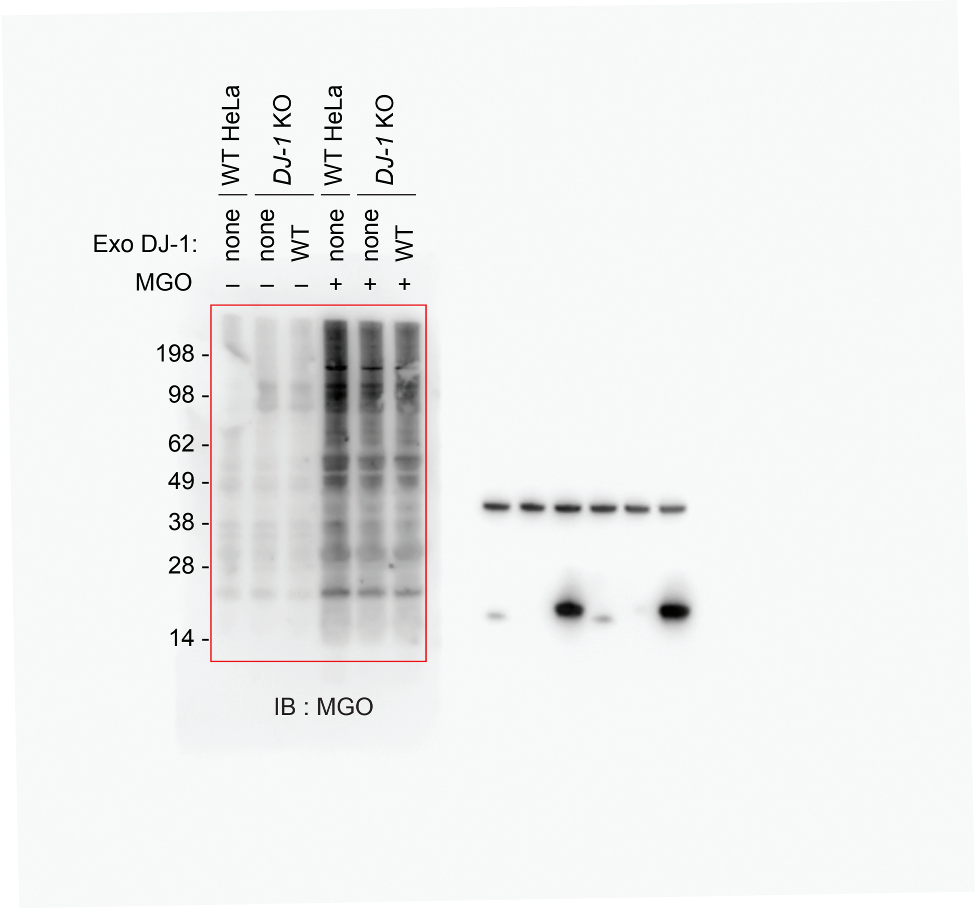

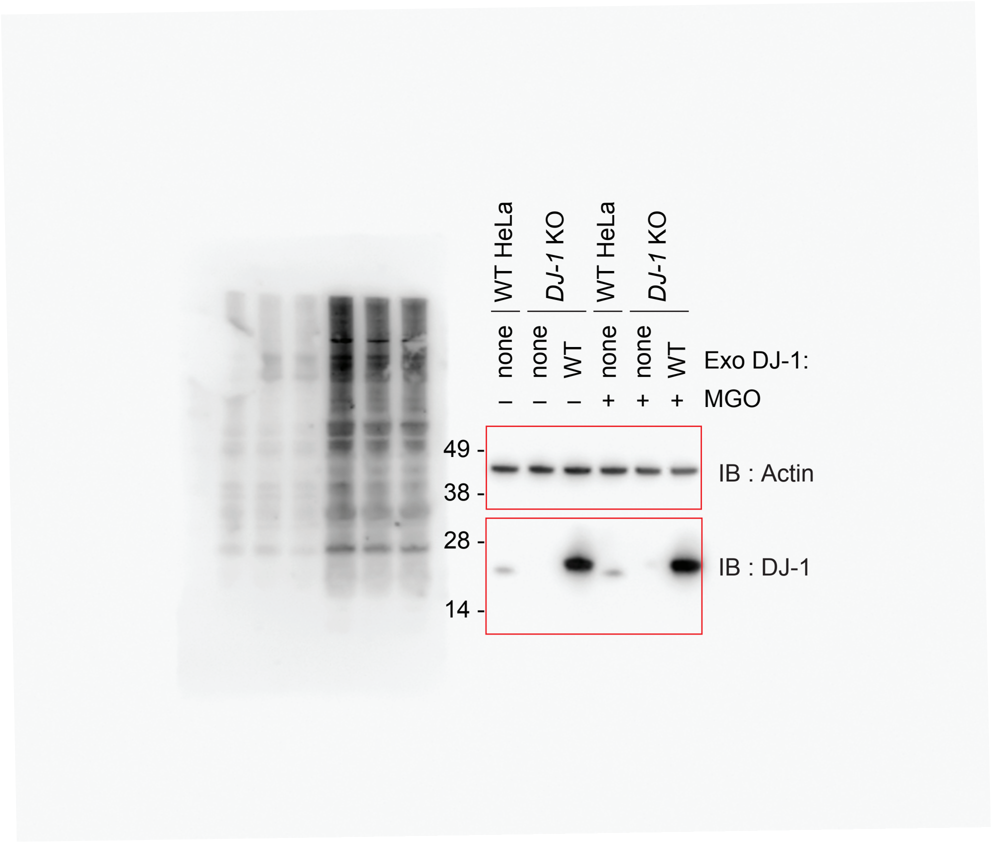

Supplement: SourceData F9 — is the source file for Fig. 9. [file jcb_202411078_sourcedataf9.pdf]
